# Potential of mineral-solubilizing bacteria for physiology and growth promotion of *Chenopodium quinoa* Willd

**DOI:** 10.3389/fpls.2022.1004833

**Published:** 2022-10-10

**Authors:** Ejaz Rafique, Muhammad Zahid Mumtaz, Inam Ullah, Aneela Rehman, Kamal Ahmad Qureshi, Muhammad Kamran, Mujaddad Ur Rehman, Mariusz Jaremko, Muneefah Abdullah Alenezi

**Affiliations:** ^1^ Institute of Molecular Biology and Biotechnology, The University of Lahore, Lahore, Pakistan; ^2^ Department of Microbiology, Abbottabad University of Science and Technology, Abbottabad, Pakistan; ^3^ Department of Pharmaceutics, Unaizah College of Pharmacy, Qassim University, Unaizah, Saudi Arabia; ^4^ School of Agriculture, Food and Wine, The University of Adelaide, Adelaide, SA, Australia; ^5^ Smart-Health Initiative (SHI) and Red Sea Research Center (RSRC), Division of Biological and Environmental Sciences and Engineering (BESE), King Abdullah University of Science and Technology (KAUST), Thuwal, Saudi Arabia; ^6^ Department of Biology, Faculty of Science, University of Tabuk, Tabuk, Saudi Arabia

**Keywords:** *Chenopodium quinoa*, Indole-3-acetic acid, minerals solubilization, phosphate solubilization, *Pontibacter* spp., *Pseudomonas* spp., 16S rRNA sequencing

## Abstract

Nutrient deficiency in wild plant species, including quinoa (*Chenopodium quinoa* Willd), can be overcome by applying mineral-solubilizing bacteria. Quinoa is a gluten-free, nutritious food crop with unique protein content. The present study aimed to characterize mineral-solubilizing rhizobacterial strains and to evaluate their plant growth-promoting potential in quinoa seedlings. More than sixty rhizobacterial strains were isolated from the quinoa rhizosphere and found eighteen strains to be strong phosphate solubilizers. Most of these bacterial strains showed zinc solubilization, and more than 80% of strains could solubilize manganese. The selected strains were identified as *Bacillus altitudinis* Cq-3, *Pseudomonas flexibilis* Cq-32, *Bacillus pumilus* Cq-35, *Pseudomonas furukawaii* Cq-40, *Pontibacter lucknowensis* Cq-48, and *Ensifer* sp. Cq-51 through 16S rRNA partial gene sequencing. Mainly, these strains showed the production of organic acids, including malic, gluconic, tartaric, ascorbic, lactic, and oxalic acids in insoluble phosphorus amended broth. All strains showed production of gluconic acids, while half of the strains could produce malic, ascorbic, lactic, and oxalic acids. These strains demonstrated the production of indole-3-acetic acid in the presence as well as in the absence of L-tryptophan. The bacterial strains also demonstrated their ability to promote growth and yield attributes, including shoot length, root length, leave numbers, root and shoot dry biomass, spike length, and spikes numbers of quinoa in pots and field trials. Increased physiological attributes, including relative humidity, quantum flux, diffusive resistance, and transpiration rate, were observed due to inoculation with mineral solubilizing bacterial strains under field conditions. *P. lucknowensis* Cq-48, followed by *P. flexibilis* Cq-32, and *P. furukawaii* Cq-40 showed promising results to promote growth, yield, and physiological attributes. The multi-traits characteristics and plant growth-promoting ability in the tested bacterial strains could provide an opportunity for formulating biofertilizers that could promote wild quinoa growth and physiology.

## Introduction

Quinoa (*Chenopodium quinoa* Willd) is a nutritious food crop farmed in South America for thousands of years. It is a significant food crop in the Andean mountains, but recently, its demand has increased in the United States, Europe, and Asia (Jacobsen, 2003). It has unique protein content and a high concentration of various vitamins and minerals (Cauda et al., 2013; Al-Barakah and Sohaib, 2019). It is one of the crops intended to offer food security in the coming times (Jaikishun et al., 2019; Maliro et al., 2021). Primarily, it is used in cooking, baking, and preparing gluten-free products, animal feed, green fodder, and pellets. It is used to prepare modified food items, including breakfast cereals, pasta, pastries, fermented beverage, industrial usage of carbohydrates, protein, saponin, and a game cover crop (Jacobsen, 2003; Urquizo et al., 2017; Tan, 2020). Quinoa grains are gluten-free, which is beneficial for sensitive people having lactose intolerance, can provide nutrition to high-activity athletes and women at risk of osteoporosis, and possess therapeutic properties for diabetes, dyslipidemia, obesity, anemia, and celiac disease (Navruz-Varli and Sanlier, 2016; Vilcacundo and Hernández-Ledesma, 2017; Guo et al., 2021).

Current farming practices largely depend on chemical fertilizers, which cause detrimental effects on crop products and consumers (Suyal et al., 2016). Indiscriminate application of chemical fertilizers causes food contamination and soil toxicity and deteriorates the physicochemical properties of soil (Alori and Fawole, 2017). The growing knowledge of health risks associated with eating low-quality crops has prompted a search for new and improved technologies to enhance crop quantity and quality without harming human health (Alori and Babalola, 2018). Microbial biofertilizers are a viable alternative to chemical inputs, promote plant development, and are involved in the biocontrol of phytopathogen (Khalid et al., 2017). They consist of plant growth-promoting microorganisms, which play a dynamic role in the accessibility of various minerals, especially nitrogen, phosphorus (P), potassium (K), zinc (Zn), and manganese (Mn) (Lalitha, 2017). Biofertilizers are the primary component of integrated nutrient management, resulting in sustainability (Kour et al., 2020b). These could be cost-effective inputs to enhance crop productivity by reducing fertilizer application and, ultimately, getting more nutrients from the soil. They are composed of living cells of efficient microbial strains that increase nutrient uptake in plants due to their associations with the rhizosphere (Mącik et al., 2020; Mumtaz et al., 2022).

Minerals are essential macronutrients and micronutrients that play a vital role in plant growth and development (Soetan et al., 2010). It is present in variable concentrations in the soil. However, many minerals’ solubilities are very low and unavailable to plants (Penn and Camberato, 2019). Mineral solubilizing bacteria (MSB) showed their ability to convert the insoluble form of minerals, including P, K, and Zn, to a soluble form and promote its uptake in plants (Divjot et al., 2021; Maharana and Dhal, 2022). This conversion is accomplished by acidification, exchange reactions, chelation, and the production of acid phosphatases and phytases (Kumar et al., 2013). These MSB also produce various secondary metabolites, including phytohormones (indole-3-acetic acid) and siderophores, contributing to plant productivity (Hariprasad and Niranjana, 2009). Different bacterial genera including *Achromobacter*, *Acinetobacter*, *Aeromonas*, *Anabaena*, *Bacillus*, *Brevibacterium*, *Burkholderia*, *Calothrix*, *Corynebacterium*, *Erwinia*, *Flavobacterium*, *Escherichia*, *Micrococcus*, *Mycobacterium*, *Nostoc*, *Paenibacillus*, *Pseudomonas*, *Rhodococcus*, *Sarcina*, *Scytonema*, *Serratia*, *Tolypothrix*, and *Xanthomonas* were isolated from soil and rhizosphere of various crops exhibited the mineral-solubilizing ability (Mishra et al., 2014; Zhang et al., 2017; Leite et al., 2018; Martínez-Gallegos et al., 2018; Singh et al., 2019; Divjot et al., 2021; Amy et al., 2022).

A limited number of studies on the bacterial interaction with quinoa were reported by Chumpitaz-Segovia et al. (2020); Mahdi et al. (2020); Yang et al. (2020); Yanez-Yazlle et al. (2021), and Testen et al. (2022). Mahdi et al. (2020) reported plant growth-promoting (PGP) characteristics, including IAA, production of siderophores, extracellular enzymes, ammonia, and hydrogen cyanide in PSB strains *Bacillus licheniformis* QA1 and *Enterobacter asburiae* QF11 isolated from quinoa rhizospheric soil. Thus, the objectives of the current study were: to isolate and screen the ability of rhizospheric strains from the quinoa rhizosphere for mineral solubilization, to characterize the potential MSB strains for *in vitro* plant growth-promoting attributes, to identify selected MSB strains based on 16S rRNA gene sequencing, and to evaluate the ability of MSB strains to promote quinoa growth in pot and field trails.

## Materials and methods

### Isolation of rhizobacteria

Rhizospheric samples of *C. quinoa* were collected from the University of Lahore (UOL) research area at 31.5204° N latitude, 74.3587° E longitude, and 217 m elevation. Samples were placed individually in sterile plastic bags and brought to the Laboratory of Microbial Biotechnology, Institute of Molecular Biology and Biotechnology (IMBB), UOL, Pakistan, under low temperature. Rhizospheric bacterial isolates were isolated from rhizospheric soil on a Luria Bertani (LB) agar medium composed of tryptone (10 g L^-1^), yeast extract (5.0 g L^-1^), NaCl (5.0 g L^-1^), and agar (15.0 g L^-1^) through serial dilution method as described by Somasegaran and Hoben (1994). The inoculated agar plates were incubated at 28 ± 1°C till the appearance of bacterial colonies. Individual colonies were picked and streaked on LB agar plates for purification. Single colonies were repeatedly re-streaked till the purified cultures were obtained. The purified bacterial cultures were preserved in 50% sterile glycerol stock at -20°C until further characterization.

### Determination of phosphate solubilization

Rhizobacterial isolates were characterized for phosphate solubilization using Pikovskaya (PVK) agar medium, as reported by Pikovskaya (1948). Bacterial strains with diverse colony morphology were inoculated in the center of PVK agar medium with the help of inoculating loop in triplicate and placed at 30 ± 1°C for seven days. The diameter of the halo zone and colony growth were measured, and phosphorus solubilization index (PSI) and solubilization efficiency (PSE) were determined using formulas described by Vazquez et al. (2000). Quantitative phosphate solubilization was determined by using the Pikovskaya broth medium in triplicate. The freshly grown colonies of bacterial strains were inoculated in Pikovskaya broth medium and kept on an orbital shaker at 100 rpm and 30 ± 1°C for ten days. The cultures were centrifuged at 10,000×*g* rpm for 5 min. The supernatant was filtered through a 0.22 µm filter (Millipore, USA). The solubilized phosphorus contents in culture filtrate were determined by adopting the method of Ryan et al. (2001). The production of organic acids in response to tri-calcium phosphate was determined by injecting culture filtrate in high-performance liquid chromatography (HPLC) equipped with Turbochrom Software (Perkin Elmer, USA) and a C-18 column. The flow rate was kept at 0.6 mL min^-1^, and used mobile phase was methanol and water (30:70 v/v). The remaining conditions were similar, as Mumtaz et al. (2019) reported. The peaks of unknown samples were compared to the standard organic acids, including gluconic acid, lactic acid, malic acid, tartaric acid, acetic acid, and citric acid, as described by Park et al. (2010).

### Zn and Mn solubilization assay

The selected PSB strains were screened for Zn solubilization as Fasim et al. (2002) described. The bacterial strains were spot inoculated on tris-minimal salt agar, amended with 1% zinc oxide, and incubated at 30 ± 1°C for seven days to observe the clear halo zone (Mumtaz et al., 2017). The Zn solubilizing index (ZSI) and Zn solubilizing efficiency (ZSE) were calculated by following the method of Vazquez et al. (2000) and Mumtaz et al. (2017). These strains were also screened for Mn solubilization by growing bacterial strains on nutrient agar amended with MnO_2_ (50 mM) at 30 ± 1°C for 72 h (Sanket et al., 2017). After incubation, Mn amended nutrient agar plates were flooded with iodine solution as an indicator to observe a clear halo zone (Ijaz et al., 2021). The clear halo zone and bacterial growth zone diameters were recorded through meter rod, and Mn solubilization index (MSI) and Mn solubilization efficiency (MSE) were calculated by following formulas reported by Ijaz et al. (2021).

### Determination of indole acetic acid

The selected MSB strains were tested for indole-3-acid (IAA) production by inoculating the LB broth amended with and without L- tryptophan (100 mg L^-1^) incubated at 30 ± 1°C for three days. After incubation, the supernatant was obtained by centrifuging the broth culture at 12000×*g* for 10 min. The supernatant of each bacterial culture was mixed with 100 µl of Salkowski reagent in a 1:1 ratio. The tubes were kept in the dark and visualized after 60 min for the development of color. The color intensity was determined at 530 nm help of a spectrophotometer. Auxin’s production by bacterial cultures in the presence and absence of L-tryptophan was estimated by plotting the standard curve of reading from the optical density of the standards solution.

### Identification of rhizobacterial strains through 16S rRNA gene sequencing

The 16S rRNA gene was amplified, sequenced, and analyzed through bioinformatics to identify MSB strains. The genomic DNA of bacterial strains was extracted with slight modifications by cetyl trimethyl ammonium bromide (CTAB; Wilson, 2001). Overnight grown bacterial cultures in LB medium at 30 ± 1°C were used for DNA extraction. Cells harvested by centrifugation at 12000 x *g* were re-suspended in 567 µL of tris-EDTA buffer (tris 10 mM; EDTA 1 mM), lysed with the 3 µL of proteinase-K, and 30 µL of 10% sodium dodecyl sulfate, followed by incubation at 30°C for an hour to allow complete lysis. The 100 µL of 5 M NaCl and 80 µL of CTAB extraction buffer (CTAB 10%; NaCl 0.7 M) was added, and the lysate was mixed thoroughly and incubated at 65°C for 10 min. DNA was purified by sequential phenol, phenol-chloroform, and chloroform extractions, followed by isopropanol precipitation. The pellets were washed with 70% ethanol and re-suspended in 100 µL of a tris-EDTA buffer. The samples were stored at -20°C until use.

The 16S rRNA gene of extracted genomic DNA was amplified through PCR amplification of the genes as described by Katsivela et al. (1999) using universal forward 9F (5′-GAGTTTGATCCTGGCTCAG-3′) and reverse primers 1510R (5′-GGCTACCTTGTTACGA-3′). Amplified PCR products of 16S rRNA were separated on 1% agarose gel in 0.5X Tris-EDTA buffer containing 2 µL ethidium bromide. The ladder λ Hind-III was used as a size marker. The gel was viewed under UV light and photographed using a gel documentation system. Amplified PCR products of full-length 16S rRNA genes were purified using a PCR purification kit (QIAGEN) according to the standard protocol recommended by the manufacturer (Laghari et al., 2010). The sequence results obtained were blasted through the DNA data bank of Japan (DDBJ). The sequence of all the related species was retrieved to get the exact terminology of the rhizobacterial strains. Phylogenetic analysis was also carried out using the bioinformatic tool molecular evolutionary genetic analysis (MEGA) software version 7.0.26 (Tamura et al., 2007). The 16S rRNA gene sequences of identified strains were submitted DDBJ database, and accession numbers against each rhizobacterial strain were obtained.

### Pot trial

A non-sterile soil culture pot experiment was conducted to check the ability of the MSB strain’s growth and yield attributes of quinoa. Fresh bacterial cultures were prepared in nutrient broth under shaking (100 rpm) conditions at 30 ± 1°C for two days. Quinoa seeds of a variety NARC-9 were soaked in the 10^-5^ CFU bacterial inoculum for half an hour. The uninoculated control treatment was prepared by soaking quinoa seeds in two days old nutrient broth without bacterial inoculation. This trial was conducted at Latitude: 31.39N, Longitude: 74.24E, and 206 meters above sea level under natural climatic conditions. The study area has a semi-arid climate characterized by foggy winter, pleasant spring, summer with dust, rainstorms, heatwave, cool winters, rainy monsoon, and dry autumn; the hottest month is June, and the coolest month is January with dense fog. The current experiment was conducted in mid of November during the winter season.

The soil used for the pot trial was collected from a research field and analyzed for physicochemical characteristics using the standard method of Ryan et al. (2001) and revealed sandy loam texture, 8.10 pH, 0.47 dS m^-1^ electrical conductivity, 0.29% organic matter, 0.02% total N, 6.0 mg kg^-1^ available P, and 176 mg kg^-1^ extractable K. Earthen pots were filled with 5 kg of non-sterilized sieved (2 mm) soil. A total of eight quinoa seeds inoculated with respective bacterial strains were sown in each pot, and three quinoa seedlings after germination were maintained in each pot. The pots were placed in a wire-house under natural environmental conditions in a completely randomized design (CRD), having three replications. The N (70 kg ha^-1^), P (40 kg ha^-1^), and K (30 kg ha^-1^) were applied in terms of urea, diammonium phosphate, and sulfate of potash. The total dose of P, K, and half dose of N was used as basal dose, and the remaining half of N was applied after one month of germination. The pots were irrigated before sowing of seeds, and after germination, pots were irrigated four times at the two-leave stage, tillering, booting, and flowering stages. The weeds were manually pulled out by hand. Plant height, root length, and panicle length were observed at physiological maturity through a meter rod. The number of leaves and panicles in each plant was manually counted. Shoot and root were shade dried and weighted through weight balance.

### Field experiment

A field experiment was conducted to evaluate the effects of plant growth-promoting MSB strains to increase quinoa growth, physiology, and yield attributes. The bacterial cultures of 48 h were prepared in nutrient broth, and the cell pellet was obtained through centrifugation at 10000×*g*. The cell pellet was washed and re-suspended in 100 mL saline solution (0.85%). Seeds were added to the cell suspension and autoclaved powdered filter-mud as carrier material. The uninoculated control seeds were coated with autoclaved filter-mud and saline solution without bacterial cell pellet. This experiment was conducted adjacent to the pot trial at the same place and same time. The soil of the field experiment was sandy loam texture, 8.10 pH, 0.28 dS m^-1^ electrical conductivity, 0.88% organic matter, 0.05% total N, 4.6 mg kg^-1^ available P, and 169 mg kg^-1^ extractable K. The field was ploughed twice, and fine seedbeds were prepared through ridger. With three replications, the coated quinoa seeds were sown under sufficient moisture conditions in Randomized Complete Block Design (RCBD). The space between plants and rows was maintained at 20 cm and 75 cm, respectively. The doses and sources of N, P, and K nutrients were the same as reported in the pot trial. After seeds germination, plots were irrigated three times before flowering and one time at the flowering stage. The weeds were removed through a manual method. At the booting stage, physiological attributes, including relative humidity, quantum reflux, diffusive resistance, and transpiration rate, were measured by a leaf porometer in a steady state during morning hours when the sky was cleared. The porometer was calibrated to ensure accurate readings before the measurements. Fresh, fully developed leaves from the top of randomly selected plants were used to record the physiological attributes. The operating environment for the porometer was 5-30°C and 10-70% relative humidity. At maturity, plants were harvested, and plant growth attributes, including plant height, root length, panicle length, number of leaves, number of panicles, shoot dry weight, and root dry weight, were recorded.

### Statistical analysis

The data from *in vitro* assay, including solubilization of P, Zn, Mn, and production of IAA, were analyzed through a one-way analysis of variance (ANOVA) in CRD arrangement. One-way ANOVA in pot and field trials (*in vivo* experiments) was employed using CRD and RCBD design, respectively. The obtained means of three replications from all *in vitro* experiments were subjected to the Tukey test, while *in vivo* experiments were analyzed through the least significant difference (LSD) test at 5% probability (Steel et al., 1997). The mean values were compared by calculating standard error in Microsoft Excel-2019 and alphabetical letters, demonstrating values sharing the same letter do not differ significantly (*P ≤* 0.05).

## Results

### Isolation of rhizobacteria

More than sixty bacterial strains were isolated from quinoa rhizospheric soil and tested for phosphate-solubilization PVK agar medium amended with tri-calcium phosphate. Eighteen rhizobacterial strains enlisted in **Table 1** showed solubilization of P. The solubilization zone diameter ranged from 12.00 ± 0.88 mm to 25.66 ± 0.76 mm, whereas the PSB strain growth diameter ranged from 6.00 ± 0.22 mm to 9.67 ± 0.57 mm. A maximum phosphate-solubilization zone diameter of 25.66 ± 0.76 mm followed by 24.00 ± 0.66 mm was reported by strains Cq-51 and Cq-40, respectively. The diameter of spot inoculated phosphate-solubilizing bacterial growth was highest in the case of Cq-15 (9.67 ± 0.57 mm) and Cq-51 (9.66 ± 0.57 mm). Strain Cq-31 reported a minimum phosphate-solubilization zone diameter of 12.00 ± 0.88 mm, while 6.00 ± 0.22 mm of the phosphate-solubilization growth zone of strain Cq-10 was the lowest. Strain Cq-10 also demonstrated lowest phosphate-solubilization index (2.72 ± 0.31) and phosphate-solubilization efficiency (172.62 ± 4.16%). A maximum phosphate-solubilization index of 4.00 ± 0.33 was reported by strain Cq-40, followed by strain Cq-32 having a 3.90 ± 0.38 phosphate-solubilization index. Strain Cq-40 and Cq-32 reported maximum phosphate-solubilization efficiency of 300.0 ± 10.22% and 290.48 ± 9.21%, respectively. A maximum quantitative phosphate-solubilized concentration of 68.43 ± 1.51 mg kg^-1^ was reported by strain Cq-48, followed by strain Cq-40 having 64.74 ± 1.47 mg kg^-1^ (**Table 1**). Strain Cq-13 reported minimum phosphorus solubilized concentration of 33.06 ± 1.19 mg kg^-1^.

**Table 1:** Phosphate solubilization by rhizobacterial strains isolated from quinoa rhizosphere.

Strains	PSZ (mm)	BG (mm)	PSI	PSE (%)	PSC (ppm)
Cq-3	19.00 ± 1.15 b-e	8.00 ± 0.30 abc	3.37 ± 0.37 a-d	237.90 ± 7.40 a-d	54.04 ± 1.63 cd
Cq-10	13.33 ± 0.57 efg	7.66 ± 0.33 abc	2.72 ± 0.31 d	172.62 ± 4.16 d	NT
Cq-13	14.00 ± 0.57 e-g	6.00 ± 0.22 c	3.36 ± 0.27 a-d	236.98 ± 11.98 a-d	33.06 ± 1.19 g
Cq-15	21.66 ± 0.57 a-d	9.67 ± 0.57 a	3.24 ± 0.46 a-d	224.07 ± 11.60 a-d	44.79 ± 1.41 e
Cq-28	13.00 ± 0.76 fg	7.33 ± 0.32 abc	2.82 ± 0.37 cd	181.94 ± 7.40 cd	NT
Cq-31	12.00 ± 0.88 g	6.66 ± 0.10 bc	2.82 ± 0.09 cd	182.14 ± 9.79 cd	NT
Cq-32	21.33 ± 1.45 a-d	7.33 ± 0.33 abc	3.90 ± 0.38 ab	290.48 ± 9.21 ab	57.59 ± 1.01 c
Cq-34	17.00 ± 0.64 d-g	7.33 ± 0.36 abc	3.32 ± 0.33 a-d	231.55 ± 9.60 a-d	34.62 ± 1.22 g
Cq-35	21.66 ± 0.66 a-d	8.66 ± 0.33 ab	3.51 ± 0.23 a-d	251.85 ± 12.91 a-d	45.93 ± 1.48 e
Cq-40	24.00 ± 0.66 ab	8.00 ± 0.33 abc	4.00 ± 0.33 a	300.0 ± 10.22 a	64.74 ± 1.47 b
Cq-41	14.00 ± 0.63 e-g	6.00 ± 0.30 c	3.37 ± 0.20 a-d	236.98 ± 12.13 a-d	34.69 ± 1.30 g
Cq-45	21.67 ± 0.88 a-d	9.00 ± 0.33 ab	3.41 ± 0.26 a-d	241.94 ± 11.02 a-d	40.13 ± 0.74 f
Cq-47	13.00 ± 0.66 fg	7.33 ± 0.33 abc	2.81 ± 0.22 cd	181.94 ± 13.63 cd	NT
Cq-48	23.00 ± 0.66 abc	9.00 ± 0.57 ab	3.57 ± 0.49 abc	257.31 ± 9.52 abc	68.43 ± 1.51 a
Cq-51	25.66 ± 0.76 a	9.66 ± 0.57 a	3.65 ± 0.51 abc	265.56 ± 6.18 abc	51.43 ± 1.37 d
Cq-52	18.00 ± 0.88 c-f	8.66 ± 0.57 ab	3.07 ± 0.43 bcd	207.41 ± 17.76 bcd	44.50 ± 1.16 e
Cq-53	13.33 ± 0.60 efg	7.67 ± 0.51 abc	2.73 ± 0.44 d	176.62 ± 9.52 d	NT
Cq-55	18.33 ± 0.66 b-f	8.33 ± 0.34 abc	3.20 ± 0.27 a-d	220.37 ± 12.43 abc	42.69 ± 0.93 ef
CVC	5.80	2.60	0.84	83.94	3.62

Phosphate-solubilization by bacterial strains was observed on Pikovskya agar media amended with tricalcium phosphate after seven days of incubation at 30 ± 1°C; Phosphate solubilization index (PSI) and phosphate solubilization efficiency (PSE) were determined using diameters of phosphate solubilization zone (PSZ) and bacterial growth (BG); quantitative phosphate solubilization was estimated through inoculating the strains in tricalcium-amended Pikoskya broth media and phosphate solubilized concentration (PSC) was detected through the colorimetric method; data presented are the mean of three replications ± standard error and Tukey HSD test was performed at 5% (P ≤ 0.05) probability level; the means in a vertical line for each attributes sharing common letters were considered statistically similar to each other; NT, not tested; CVC, critical value for comparison.

### Solubilization of Zn and Mn

Phosphate solubilizing bacterial strains were screened for the qualitative solubilization of Zn and Mn, and their results are given in **Tables 2** and **3**. All the tested phosphate solubilizing bacterial strains showed solubilization of Zn except strain Cq-31. Maximum zinc solubilization zone was reported by strains Cq-40, Cq-47, Cq-51, and Cq-53, having a diameter of 21.33 ± 0.66 mm, 21.33 ± 0.57 mm, 21.33 ± 1.15 mm, and 20.33 ± 0.66 mm, respectively. The zinc solubilization zone diameter of these strains was statistically similar to each other and various other strains depicted in **Table 2**. Strain Cq-13 reported a minimum zinc solubilization zone diameter of 9.00 ± 0.57 mm. The growth diameter (9.33 ± 0.66 mm) of strain Cq-28 was more prominent, while strain Cq-15 reported a smaller bacterial growth zone diameter of 6.00 ± 0.33 mm. Strain Cq-51 reported a maximum zinc solubilization index of 3.68 ± 0.37 and zinc solubilization efficiency of 268.85 ± 17.76%. Minimum zinc solubilization index of 2.42 ± 0.40 and zinc solubilization efficiency of 142.86 ± 11.98% was reported by strain Cq-13.

**Table 2:** Solubilization of zinc by phosphate solubilizing bacterial strains isolated from quinoa rhizosphere.

Strains	ZSZ (mm)	BGZ (mm)	ZSI	ZSE (%)
Cq-3	15.00 ± 1.66 bcd	7.66 ± 0.33 a-d	2.95 ± 0.48 bc	195.83 ± 4.16 bc
Cq-10	18.00 ± 1.15 a-d	8.65 ± 0.57 ab	3.07 ± 0.54 abc	207.41 ± 7.40 abc
Cq-13	9.00 ± 0.57 e	6.33± 0.57 cd	2.42 ± 0.40 c	142.86 ± 11.98 c
Cq-15	13.00 ± 1.38 de	6.00 ± 0.33 d	3.18 ± 0.30 ab	218.89 ± 11.60 ab
Cq-28	19.66 ± 1.84 ab	9.33 ± 0.66 a	3.08 ± 0.83 abc	208.33 ± 16.91 abc
Cq-31	ND	ND	ND	ND
Cq-32	17.66 ± 0.88 a-d	9.00 ± 0.88 ab	2.96 ± 0.54 bc	196.30 ± 9.79 bc
Cq-34	18.00 ± 0.57 a-d	7.00 ± 0.57 bcd	3.59 ± 0.41 ab	259.92 ± 17.79 ab
Cq-35	18.00 ± 1.84 a-d	7.66 ± 0.33 a-d	3.35 ± 0.09 ab	235.71 ± 12.91 ab
Cq-40	21.33 ± 0.66 a	8.66 ± 0.33 ab	3.46 ± 0.29 ab	246.76 ± 12.43 ab
Cq-41	14.00 ± 1.15 cde	6.00 ± 0.57 d	3.39 ± 0.28 ab	239.37 ± 24.18 ab
Cq-45	18.00 ± 0.66 a-d	8.33 ± 0.57 abc	3.16 ± 0.48 ab	216.67 ± 11.02 ab
Cq-47	21.33 ± 0.57 a	9.00 ± 0.57 ab	3.38 ± 0.44 ab	236.15 ± 9.52 ab
Cq-48	16.66 ± 0.57 a-d	8.33 ± 0.66 abc	3.00 ± 0.41 abc	200.93 ± 13.63 abc
Cq-51	21.33 ± 1.15 a	8.00 ± 0.30 a-d	3.68 ± 0.37 a	268.85 ± 17.76 a
Cq-52	18.66 ± 0.88 abc	8.00 ± 0.23 a-d	3.34 ± 0.32 ab	234.19 ± 6.18 ab
Cq-53	20.33 ± 0.66 a	8.66 ± 0.41 ab	3.34 ± 0.31 ab	234.72 ± 11.38 ab
Cq-55	17.33 ± 1.73 a-d	8.66 ± 0.33 ab	3.00 ± 0.21 abc	200.00 ± 21.00 abc
CVC	5.25	2.32	0.72	71.65

Phosphate-solubilizing bacterial strains were incubated for zinc solubilization on tris-minimal salt agar media amended with ZnO for seven days at 30 ± 1°C; Zinc solubilization index (ZSI) and zinc solubilization efficiency (ZSE) were determined using diameters of zinc solubilization zone (ZSZ) and bacterial growth (BG); data presented are the mean of three replications ± standard error and Tukey HSD test was performed at 5% (P ≤ 0.05) probability level; the means in a vertical line for each attributes sharing common letters were considered statistically similar to each other; ND, not detected; CVC, critical value for comparison.

**Table 3:** Solubilization of manganese by phosphate solubilizing bacterial strains isolated from quinoa rhizosphere.

Strains	MSZ (mm)	BGZ (mm)	MSI	MSE (%)
Cq-3	26.66 ± 0.33 b-f	9.00 ± 0.57 bc	3.98 ± 0.38 abc	298.80 ± 20.10 abc
Cq-10	24.00 ± 0.57 efg	8.66 ± 0.30 b-e	3.77 ± 0.28 abc	277.31 ± 6.01 abc
Cq-13	24.33 ± 0.33 efg	7.66 ± 0.33 de	4.18 ± 0.21 ab	318.45 ± 12.72 ab
Cq-15	28.66 ± 0.33 bc	9.66 ± 0.31 abc	3.97 ± 0.26 abc	297.04 ± 7.03 abc
Cq-28	ND	ND	ND	ND
Cq-31	29.66 ± 0.88 b	10.33 ± 0.29 ab	3.87 ± 0.31 abc	287.27 ± 6.38 abc
Cq-32	33.66 ± 0.33 a	11.33 ± 0.33 a	3.97 ± 0.26 abc	297.47 ± 7.54 abc
Cq-34	ND	ND	ND	ND
Cq-35	27.00 ± 0.57 b-e	8.66 ± 0.35 b-e	4.12 ± 0.26 abc	312.04 ± 7.23 abc
Cq-40	24.33 ± 0.33 efg	8.00 ± 0.57 cde	4.06 ± 0.27 abc	246.76 ± 14.88 abc
Cq-41	ND	ND	ND	ND
Cq-45	28.00 ± 0.57 bcd	8.66 ± 0.33 b-e	4.23 ± 0.26 a	323.61 ± 7.64 a
Cq-47	23.66 ± 0.88 fg	8.66 ± 0.34 b-e	3.73± 0.32 bc	273.15 ± 3.33 bc
Cq-48	25.00 ± 1.15 d-g	9.33 ± 0.30 bcd	3.67 ± 0.35 c	267.78 ± 6.51 c
Cq-51	26.00 ± 0.57 d-g	9.33 ± 0.33 bcd	3.79 ± 0.18 abc	279.63 ± 15.15 abc
Cq-52	26.00 ± 0.88 c-f	8.33 ± 0.35 cde	4.16 ± 0.30 ab	316.20 ± 4.41 ab
Cq-53	ND	ND	ND	ND
Cq-55	22.00 ± 0.57 g	7.33 ± 0.33 e	4.00 ± 0.27 abc	300.60 ± 7.73 abc
LSD	3.12	1.77	0.48	47.54

Phosphate-solubilizing bacterial strains were incubated for manganese solubilization on nutrient agar media amended with MnO_2_ for three days at 30 ± 1°C; Manganese solubilization index (MSI) and manganese solubilization efficiency (MSE) were determined using diameters of manganese solubilization zone (MSZ) and bacterial growth (BG); data presented are the mean of three replications ± standard error and Tukey HSD test was performed at 5% (P ≤ 0.05) probability level; the means in a vertical line for each attributes sharing common letters were considered statistically similar to each other; ND, not detected; CVC, critical value for comparison.

Strains Cq-28, Cq-34, Cq-41, and Cq-53 could not solubilize manganese; however, all other tested phosphate solubilizing bacterial strains were well-capable to solubilize manganese (**Table 3**). Strain Cq-32 followed by strain Cq-31 reported maximum manganese solubilization zones of 33.66 ± 0.33 mm and 29.66 ± 0.88 mm, respectively. These strains also showed higher bacterial growth zones of 11.33 ± 0.33 mm and 10.33 ± 0.29 mm, respectively. A maximum manganese solubilization index of 4.23 ± 0.26 and manganese solubilization efficiency of 323.61 ± 7.64% was reported by strain Cq-45. Strain Cq-13 and Cq-52 also reported better manganese solubilization index of 4.18 ± 0.21 and 4.16 ± 0.30, respectively, and manganese solubilization efficiency of 318.45 ± 12.72% and 316.20 ± 4.41%, respectively.

### Production of organic acids

The best six MSB strains, e.g., Cq-3, Cq-32, Cq-35, Cq-40, Cq-48, and Cq-51, can solubilize zinc and manganese were screened for production of organic acids by using HPLC. Most of these strains showed the production of malic, gluconic, tartaric, ascorbic, lactic, and oxalic acids and their detected concentration by each strain (**Table 4**). Malic acid was produced by all bacterial strains except strains Cq-3 and Cq-51. The highest malic acid concentration was produced by strain Cq-48 (12.11 ± 0.82 µg mL^-1^), followed by strain Cq-35 (11.50 ± 1.23 µg mL^-1^), while strain Cq-32 reported the lowest malic acid concentration (1.96 ± 0.07 µg mL^-1^). All the tested bacterial strains produced gluconic acid in PVK broth amended with tri-calcium phosphate. Strain Cq-3 followed by strain Cq-35 reported maximum gluconic acid concentration of 12.61 ± 1.02 µg mL^-1^ and 12.04 ± 1.02 µg mL^-1^. A minimum gluconic acid concentration of 2.46 ± 0.04 µg mL^-1^ was produced by strain Cq-51. Only strain Cq-48 reported production of tartaric acid (3.01 ± 0.52 µg mL^-1^). Ascorbic acid was created by three strains, Cq-48, Cq-35, and Cq-3 having a concentration of 1.20 ± 0.05 µg mL^-1^, 1.33 ± 0.07 µg mL^-1^, and 15.01 ± 1.12 µg mL^-1^, respectively. Strains Cq-32, Cq-40, and Cq-51 were unable to produce ascorbic acid. Strains Cq-3, Cq-40, and Cq-51 were also unable to produce lactic acid. Maximum lactic acid was produced by strain Cq-48 (13.21 ± 1.44 µg mL^-1^), followed by strains Cq-32 (7.38 ± 1.23 µg mL^-1^) and Cq-35 (3.99 ± 0.09 µg mL^-1^). Three strains, e.g., Cq-3, Cq-40, and Cq-48, reported oxalic acid production out of six tested strains. Strain Cq-3 reported a maximum oxalic acid concentration of 8.72 ± 1.32 µg mL^-1^.

**Table 4:** Production of organic acids by phosphate solubilizing bacterial strains grown in Pikovskya broth amended with tri-calcium phosphate.

Strains	Malic acid	Gluconic acid	Tartaric acid	Ascorbic acid	Lactic acid	Oxalic acid
	µg mL^-1^					
Cq-3	ND	12.61 ± 1.02 a	ND	1.20 ± 0.05 b	ND	8.72 ± 1.32 a
Cq-32	1.96 ± 0.07 c	10.79 ± 0.77 b	ND	ND	7.38 ± 1.23 b	ND
Cq-35	11.50 ± 1.23 a	12.04 ± 1.70 a	ND	1.33 ± 0.07 b	3.99 ± 0.09 c	ND
Cq-40	5.25 ± 0.12 b	3.47 ± 0.05 d	ND	ND	ND	1.97 ± 0.21 b
Cq-48	12.11 ± 0.82 a	5.00 ± 0.21 c	3.01 ± 0.52 a	15.01 ± 1.12 a	13.21 ± 1.44 a	2.95 ± 0.07 b
Cq-51	ND	2.46 ± 0.04 d	ND	ND	ND	ND
CVC	1.05	1.51	0.37	0.79	1.34	0.95

The production of organic acids by phosphate-solubilizing bacterial strains was determined by inoculating the strains in Pikovskya broth amended with tri-calcium phosphate. The cultures were incubated at 30 ± 1°C for ten days. Organic acids were determined through high-performance liquid chromatography; the data presented are the mean of three replications ± standard error, and the Tukey HSD test was performed at 5% (P ≤ 0.05) probability level; the means in a vertical line for each attributes sharing common letters were considered statistically similar to each other; ND, not detected; CVC, critical value for comparison.

### Production of indole acetic acid

Indole acetic acid production by selected MSB strains was evaluated in the presence and absence of L-tryptophan. The tested strains showed indole acetic acid production both in the presence and absence of L-tryptophan; however, indole acetic acid production was higher in the presence of L-tryptophan (**Table 5**). The most increased production of indole acetic acid in the presence of L-tryptophan was observed in strains Cq-48 (19.76 ± 1.35 μg mL^-1^) and Cq-51 (18.72 ± 1.31 μg mL^-1^). Strain Cq-3 reported the lowest production of indole acetic acid in the absence of L-tryptophan (3.83 ± 0.56 μg mL^-1^) and the presence of L-tryptophan (7.93 ± 0.48 μg mL^-1^). In the absence of L-tryptophan, maximum indole acetic acid production was observed by strain Cq-48 (8.06 ± 0.34 μg mL^-1^) followed by Cq-51 (5.76 ± 0.20 μg mL^-1^).

**Table 5:** Production of indole-3-acetic acid by phosphate solubilizing bacterial strains isolated from quinoa rhizosphere.

Strains	Without L-tryptophan (µg mL^-1^)	With L-tryptophan (µg mL^-1^)
*Bacillus altitudinis* Cq-3	3.83 ± 0.56 c	7.93 ± 0.48 c
*Pseudomonas flexibilis* Cq-32	4.37 ± 0.28 c	9.49 ± 0.66 c
*Bacillus pumilus* Cq-35	4.46 ± 0.26 c	9.10 ± 0.74 c
*Pseudomonas furukawaii* Cq-40	4.47 ± 0.19 c	13.13 ± 1.04 b
*Pontibacter lucknowensis* Cq-48	8.06 ± 0.34 a	19.76 ± 1.35 a
*Ensifer* sp. Cq-51	5.76 ± 0.20 b	18.72 ± 1.31 a
CVC	1.04	1.64

The indole-3-acetic acid production in the presence and absence of L-tryptophan was determined by inoculating the strains in Luria-Bertani broth amended with and without L-tryptophan for three days; the data presented are the mean of three replications ± standard error; the Tukey HSD test was performed at 5% (P ≤ 0.05) probability level; the means in a vertical line for each attributes sharing common letters were considered statistically similar to each other; CVC, critical value for comparison.

### Identification of bacterial strains

**Figure 1:**
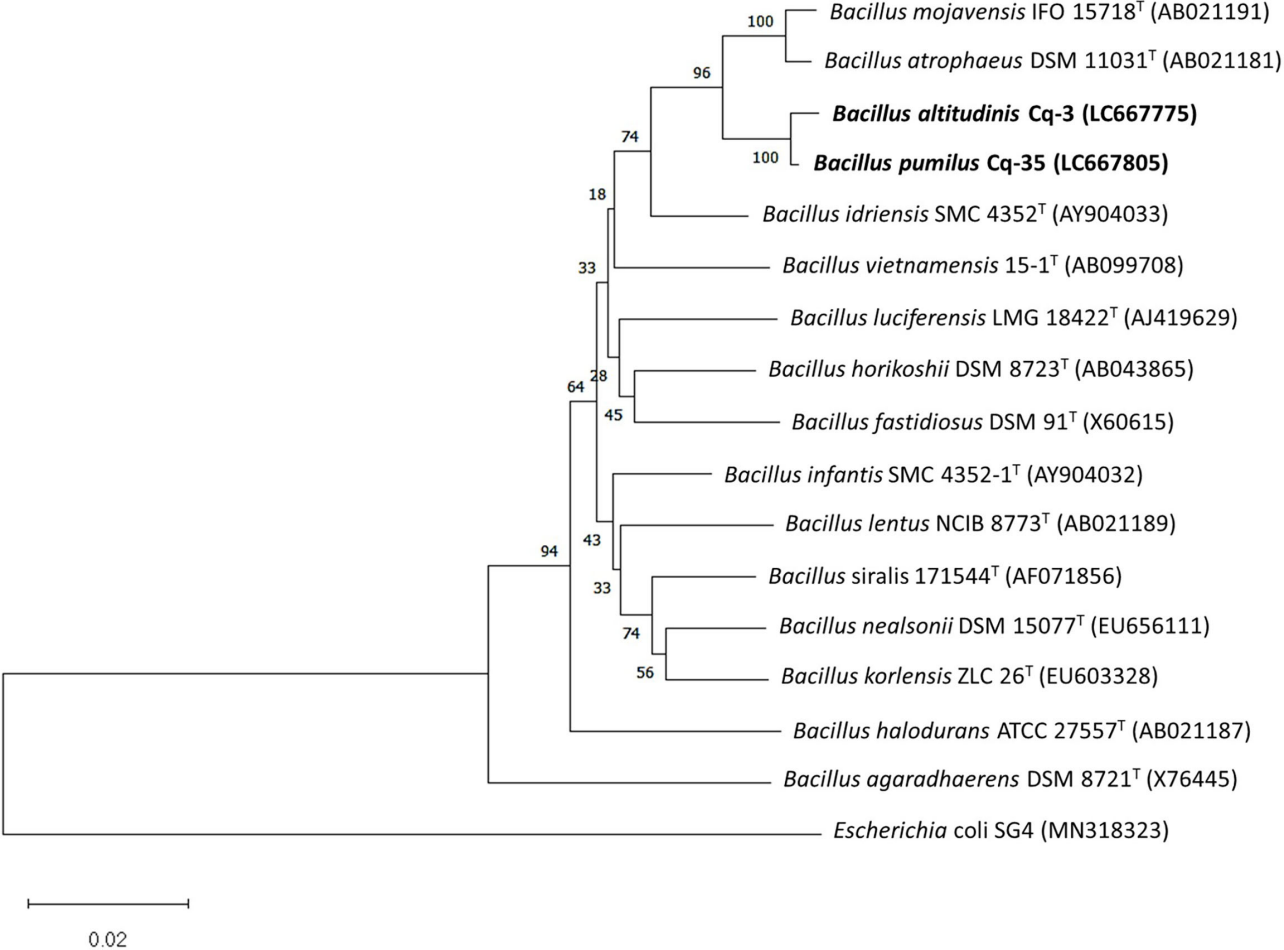
Phylogenetic tree of the strains Cq-3 and Cq-35 and related type strains within family *Bacillaceae* based on partial 16S rRNA gene sequences. The neighbor-joining algorithm constructed the tree in MEGA_X.

**Figure 2:**
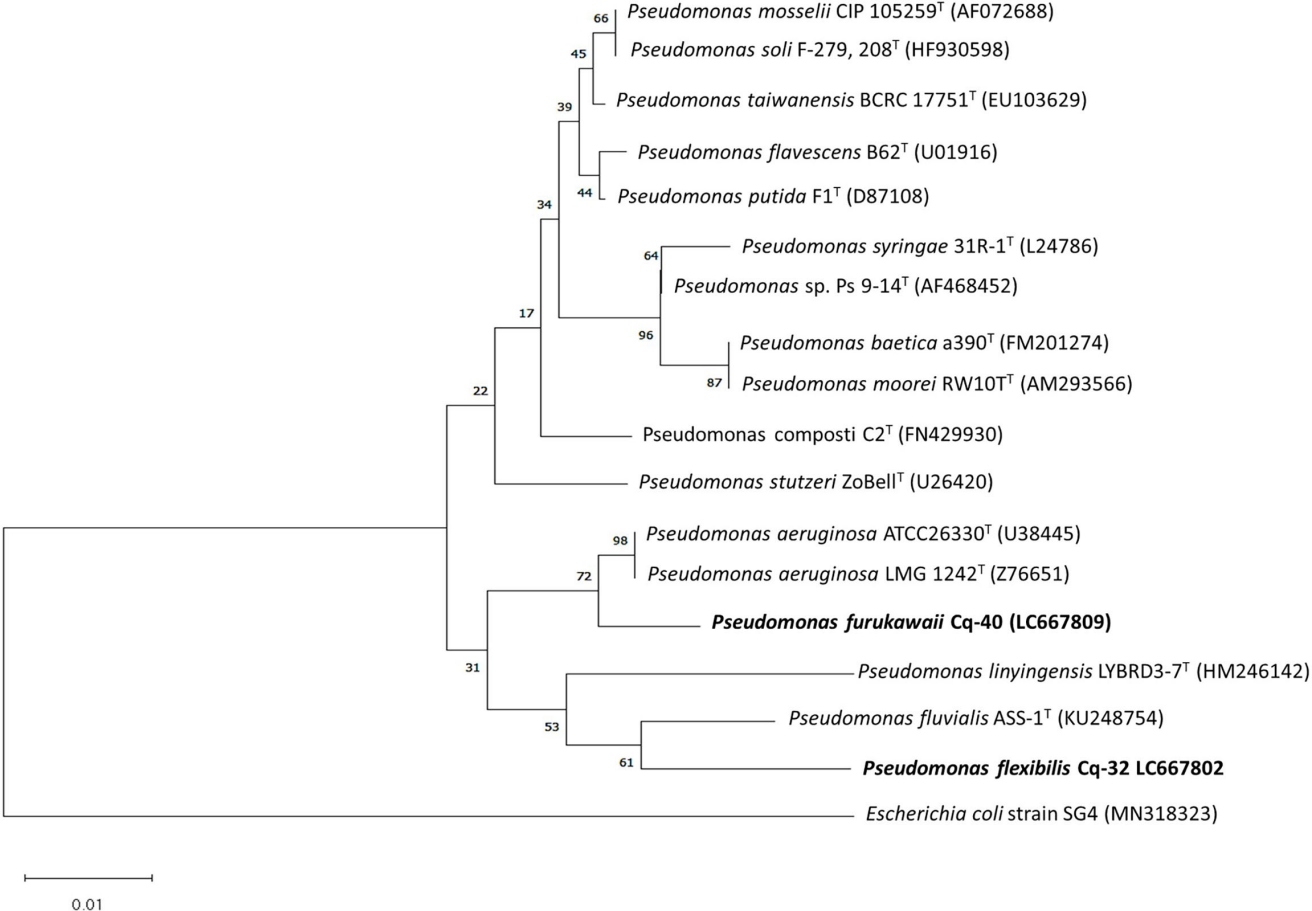
Phylogenetic tree of the strains Cq-32 and Cq-40 and related type strains within family *Pseudomonadaceae* based on partial 16S rRNA gene sequences. The neighbor-joining algorithm constructed the tree in MEGA_X.

**Figure 3:**
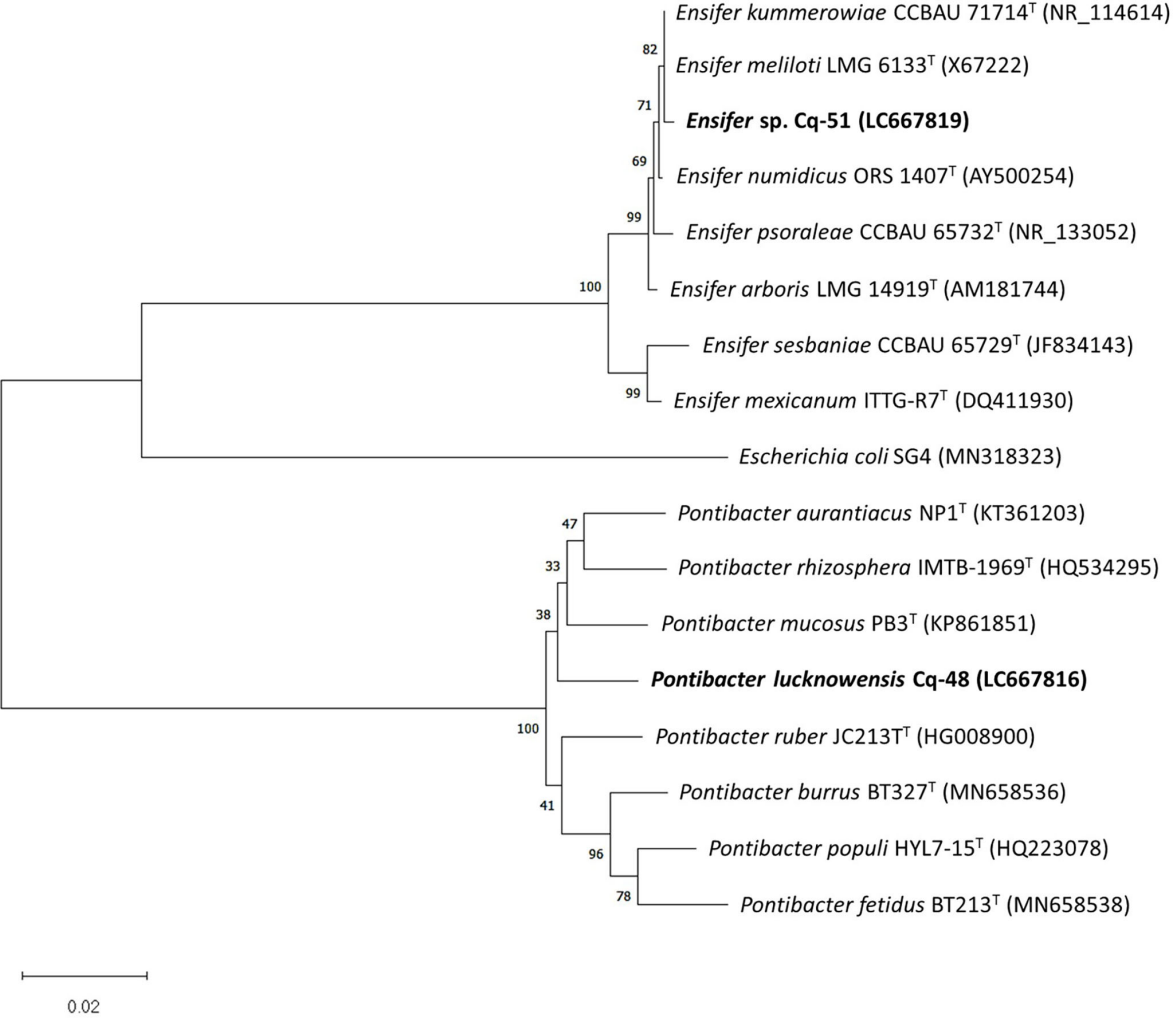
Phylogenetic tree of the strains Cq-32 and Cq-40 and related type strains within families of *Rhizobiaceae* and *Cytophagaceae* based on partial 16S rRNA gene sequences. The neighbor-joining algorithm constructed the tree in MEGA_X.

The selected strains, e.g., Cq-3, Cq-32, Cq-35, Cq-40, Cq-48, and Cq-51, were identified through 16S rRNA sequences. The obtained sequences were blasted on NCBI nucleotides blast service and identified by closely related species. **Table 6** elucidates the identified strains’ accession number, the closely related species, and their similarity index. These strains were identified as *Bacillus altitudinis* Cq-3, *Pseudomonas flexibilis* Cq-32, *Bacillus pumilus* Cq-35, *Pseudomonas furukawaii* Cq-40, *Pontibacter lucknowensis* Cq-48, and *Ensifer* sp. Cq-51. The obtained accession number of identified strains were LC667775 (*B. altitudinis* Cq-3), LC667802 (*P. flexibilis* Cq-32), LC667805 (*B. pumilus* Cq-35), LC667809 (*P. furukawaii* Cq-40), LC667816 (*P. lucknowensis* Cq-48), and LC667819 (*Ensifer* sp. Cq-51). Phylogenetic analysis was performed by comparing the tested strains with closely related type strains of the related genus. Phylogenic analysis of strains *B. altitudinis* Cq-3 and *B. pumilus* Cq-35 is given in **Figure 1**, while **Figure 2** represents the phylogenetic analysis of strains *P. flexibilis* Cq-32 and *P. furukawaii* Cq-40. In **Figure 3**, phylogenetic comparison for strains *P. lucknowensis* Cq-48 and *Ensifer* sp. Cq-51 was performed. The morphological characters of these strains are given in **Table S1**. All the strains were rod-shaped except *P. flexibilis* Cq-32. Half of the strains were Gram-positive, and half were Gram-negative. The selected strains showed variable colonies’ appearance. The colony of strain *B. altitudinis* Cq-3 was fuzzy white, while *P. flexibilis* Cq-32 colony appearance was reddish. *B. pumilus* Cq-35, *P. furukawaii* Cq-40, and *P. lucknowensis* Cq-48 colonies appeared yellow-orange, orange, and yellow, respectively. *Ensifer* sp. Cq-51 colony was of peach appearance.

**Table 6:** Identification of *Chenopodium quinoa-*associated bacterial strains based on 16S rRNA gene sequence analysis.

Strain code	Identified species	Accession No.	Closest match (accession number)	Similarity
Cq-3	*Bacillus altitudinis*	LC667775	*Bacillus altitudinis* strain 41KF2bT.10 (MN543810)	99.7%
Cq-32	*Pseudomonas flexibilis*	LC667802	*Pseudomonas flexibilis* strain ATCC 29606 (NR_104838)	99.9%
Cq-35	*Bacillus pumilus*	LC667805	*Bacillus pumilus* strain Agri-15 (MT102723)	100.0%
Cq-40	*Pseudomonas furukawaii*	LC667809	*Pseudomonas furukawaii* strain RS3 (KY986923)	100.0%
Cq-48	*Pontibacter lucknowensis*	LC667816	*Pontibacter lucknowensis* strain DM9 (NR_109478)	99.1%
Cq-51	*Ensifer* sp.	LC667819	*Ensifer* sp. strain LCK9 (MN596033)	100.0%

### Promotion of quinoa growth under pot trial

The MSB strains were screened for their ability to promote the development of quinoa under a soil culture pot trial. Inoculated quinoa plants showed significantly higher plant height, leaves count, root length, shoot dry weight, root dry weight, number of spikes, and spike length (**Tables 7**, **8**). Uninoculated control reported minimum plant height, root length, leaves count, and shoot fresh and dry weights. Strain *P. lucknowensis* Cq-48 reported a significantly maximum increase of 1.54 fold in plant height compared to the uninoculated control (**Table 7**). Strains *B. altitudinis* Cq-3 and *P. flexibilis* Cq-32 also reported better growth in plant height, increasing up to 1.33 and 1.42 fold. These strains were non-significant to each other and with strains *B. pumilus* Cq-35, *P. furukawaii* Cq-40, and *Ensifer* sp. Cq-51, all of these strains were significantly different from uninoculated control. Strain *Ensifer* sp. Cq-51 reported a maximum increase of 1.55 fold in root length, which was statistically similar to strain *P. lucknowensis* Cq-48, increasing to 1.39 fold over uninoculated control (**Table 7**). The maximum leaves count was observed from strain *P. lucknowensis* Cq-48 with an increase of 2.25 fold compared to uninoculated control (**Table 7**). Strains *B. altitudinis* Cq-3 and *P. flexibilis* Cq-32 were also better at producing leaves count, increasing up to 1.74 and 1.77 fold, respectively, over uninoculated control.

**Table 7:** Promotion of plant height, root length, and leaves count of quinoa through inoculation with phosphate solubilizing bacterial strains under pot trial.

Strains	Plant height (cm)	Root length (cm)	Leaves count
Uninoculated Control	27.94 ± 1.46 c	5.84 ± 0.29 c	24.33 ± 1.76 c
*Bacillus altitudinis* Cq-3	37.25 ± 3.69 b	6.77 ± 0.84 bc	42.33 ± 2.64 b
*Pseudomonas flexibilis* Cq-32	39.79 ± 2.24 b	7.19 ± 0.73 bc	43.00 ± 1.20 b
*Bacillus pumilus* Cq-35	33.02 ± 3.69 bc	5.84 ± 0.47 c	30.66 ± 2.48 c
*Pseudomonas furukawaii* Cq-40	33.87 ± 2.93 bc	7.28 ± 0.94 bc	32.67 ± 0.88 bc
*Pontibacter lucknowensis* Cq-48	43.34 ± 1.46 a	8.12 ± 0.38 ab	54.66 ± 2.02 a
*Ensifer* sp. Cq-51	35.56 ± 3.87 bc	9.06 ± 0.75 a	26.00 ± 3.51 c
CVC	8.57	1.70	10.75

The data presented here are the mean of three replications ± standard error; the least significant difference (LSD) test was performed at a 5% (P ≤ 0.05) probability level; the means in a vertical line for each attributes sharing common letters were considered statistically similar to each other; CVC, critical value for comparison.

**Table 8:** Promotion of quinoa’s shoot weight, root weight, and spike growth through inoculation with phosphate solubilizing bacterial strains under pot trial.

Strains	Shoot dry weight (g)	Root dry weight (g)	Number of spikes	Spike length (cm)
Uninoculated Control	11.00 ± 0.57 b	2.26 ± 0.13 c	6.50 ± 0.57 c	11.00 ± 0.84 d
*Bacillus altitudinis* Cq-3	18.97 ± 6.75 ab	2.77 ± 0.18 bc	11.33 ± 1.20 ab	20.32 ± 1.46 bc
*Pseudomonas flexibilis* Cq-32	13.00 ± 0.88 b	3.40 ± 0.28 b	12.00 ± 1.15 ab	22.01 ± 0.84 ab
*Bacillus pumilus* Cq-35	14.33 ± 1.93 ab	2.63 ± 0.46 bc	10.00 ± 0.88 b	17.36 ± 1.46 c
*Pseudomonas furukawaii* Cq-40	14.67 ± 0.63 ab	2.63 ± 0.37 bc	11.33 ± 1.45 ab	20.32 ± 1.12 bc
*Pontibacter lucknowensis* Cq-48	22.66 ± 2.40 a	4.93 ± 0.53 a	13.66 ± 1.85 a	25.40 ± 1.46 a
*Ensifer* sp. Cq-51	17.00 ± 1.15 ab	5.33 ± 0.44 a	11.00 ± 0.57 ab	18.62 ± 2.24 bc
CVC	8.77	1.09	3.50	4.31

The data presented here are the mean of three replications ± standard error; the least significant difference (LSD) test was performed at a 5% (P ≤ 0.05) probability level; the means in a vertical line for each attributes sharing common letters were considered statistically similar to each other; CVC, critical value for comparison.

Strain *P. lucknowensis* Cq-48 produces maximum shoot dry weight with a 2.06 fold increase over uninoculated control (**Table 8**). This strain was similar to strains Cq-3, *B. pumilus* Cq-35, *P. furukawaii* Cq-40, and *Ensifer* sp. Cq-51; however, it was statistically different from uninoculated control. Strains *P. lucknowensis* Cq-48 and *Ensifer* sp. Cq-51 reported the maximum root dry weight with an increase of 2.18 and 2.35 fold, respectively, over uninoculated control (**Table 8**). These strains were non-significant to each other but found to be significantly different from uninoculated control. Strain *P. lucknowensis* Cq-48 demonstrated a maximum number of spikes with a 2.10-fold increase, compared to the uninoculated control (**Table 8**). This strain was similar to strains *B. altitudinis* Cq-3, *P. flexibilis* Cq-32, *P. furukawaii* Cq-40, and *Ensifer* sp. Cq-51; however, it was found statistically different from uninoculated control. Strain *P. lucknowensis* Cq-48 reported a significantly maximum spike length with a 2.31-fold increase, followed by strain *P. flexibilis* Cq-32 having a 2.00-fold increase over uninoculated control (**Table 8**). These strains were statistically similar but found statistically different from uninoculated control. Strain *P. flexibilis* Cq-32 was also statistically identical to strains *B. altitudinis* Cq-3, *P. furukawaii* Cq-40, and *Ensifer* sp. Cq-51, but these strains were statistically different from uninoculated control. Uninoculated control reported minimum root fresh and dry weight, number of spikes, and spike length.

### Promotion of physiological and growth attributes of quinoa under field conditions

**Figure 4:**
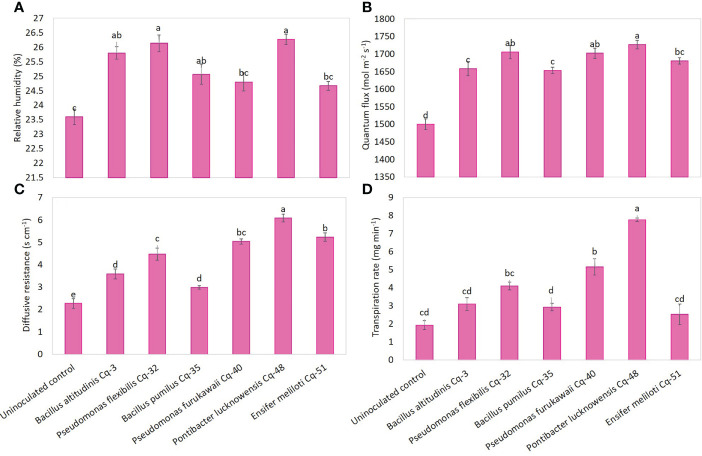
Effect of phosphate-solubilizing bacterial strains on quinoa physiological attributes in field conditions; the physiological characteristics were recorded in terms of relative humidity **(A)**, quantum flux **(B)**, diffusive resistance **(C)**, and transpiration rate **(D)**; the control had no inoculum (uninoculated control) and contains only broth; data presented are the mean of three replications along with standard error; the means sharing common letters were considered statistical similar to each other, and least significant difference (LSD) test was performed at 5% (*P* ≤ 0.05) probability level.

Under field conditions, seed-applied MSB strains significantly promoted quinoa’s physiological attributes, including relative water contents, quantum flux, diffusive resistance, and transpiration rate (**Figure 4**). Strains *P. lucknowensis* Cq-48 and *P. flexibilis* Cq-32 showed a maximum relative humidity with an increase of 1.11 and 1.10 fold over uninoculated control (**Figure 4A**). These strains were non-significant to each other and with strains *B. pumilus* Cq-35 and *B. altitudinis* Cq-3; however, they were found to be significantly different from uninoculated control. The highest increase of 1.15 fold in quantum flux was recorded due to the application with strain *P. lucknowensis* Cq-48 compared to the uninoculated control (**Figure 4B**). The *P. lucknowensis* Cq-48 was statistically similar to *P. furukawaii* Cq-40 (1.13 fold higher over uninoculated control) and *P. flexibilis* Cq-32 (1.13 fold higher over uninoculated control). Diffusive resistance was statistically higher due to strain *P. lucknowensis* Cq-48 which showed an increase of 2.69 fold compared to uninoculated control (**Figure 4C**). Strains *Ensifer* sp. Cq-51 and *P. furukawaii* Cq-40 also reported a better increase of 2.31 and 2.22 fold over uninoculated control. A maximum 4.02 fold increase in transpiration rate compared to uninoculated control was observed from *P. lucknowensis* Cq-48, followed by *P. furukawaii* Cq-40 and *P. flexibilis* Cq-32, which reported 2.67 and 2.12 fold higher transpiration rates over uninoculated control (**Figure 4D**). The lowest physiological activities in relative water contents, quantum flux, diffusive resistance, and transpiration rate were observed in the case of uninoculated control.

**Figure 5:**
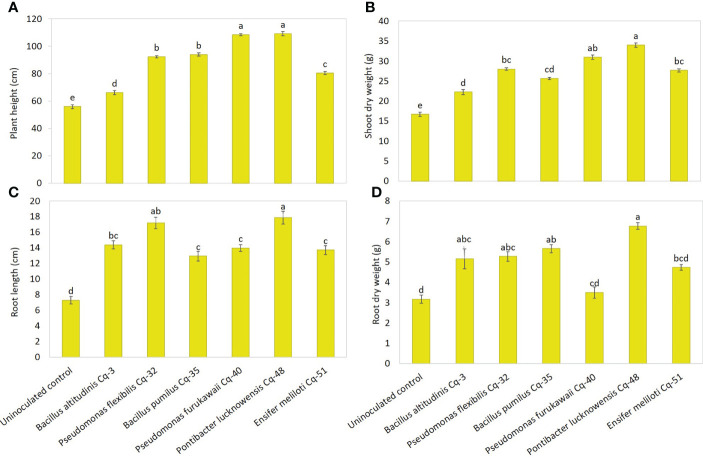
Effect of phosphate-solubilizing bacterial strains on quinoa growth attributes in field conditions; growth attributes were recorded in terms of plant height **(A)**, shoot dry weight **(B)**, root length **(C)**, and root dry weight **(D)**; the control had no inoculum (uninoculated control) and contains only broth; data presented are the mean of three replications along with standard error; the means sharing common letters were considered statistical similar to each other, and least significant difference (LSD) test was performed at 5% (*P* ≤ 0.05) probability level.

**Figure 6:**
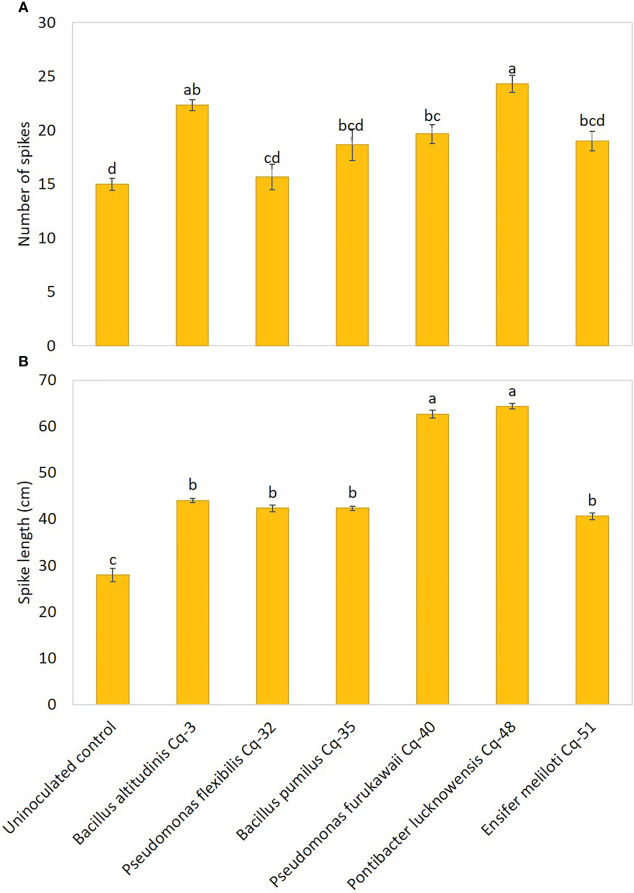
Effect of phosphate-solubilizing bacterial strains on quinoa spike count **(A)** and spike length **(B)** in field conditions; the control had no inoculum (uninoculated control) and contained only broth; data presented are the mean of three replications along with standard error; the means sharing common letters were considered statistical similar to each other, and least significant difference (LSD) test was performed at 5% (*P* ≤ 0.05) probability level.

The effect of the seed-applied MSB strains on growth attributes is demonstrated in **Figure 5**. The uninoculated control reported the lowest values for plant height, root length, shoot dry weight, and root dry weight. Strains *P. lucknowensis* Cq-48 and *P. furukawaii* Cq-40 showed the highest 1.95 and 1.94 fold increase, respectively, over uninoculated control (**Figure 5A**). These strains also reported maximum shoot dry weight with a 2.04 and 1.86 fold increase, respectively, compared to uninoculated control (**Figure 5B**). These strains were statistically similar for plant height and shoot dry weight; however, they were found significantly different from respective uninoculated control. The maximum 2.45-fold increase in root length of quinoa was observed due to inoculation with *P. lucknowensis* Cq-48 over uninoculated control (**Figure 5C**). Strain *P. flexibilis* Cq-32 also reported a better increase of 2.36 fold in root length of quinoa over uninoculated control. A maximum 2.14-fold increase over uninoculated control was observed from strain *P. lucknowensis* Cq-48 which was statistically similar to strains *B. altitudinis* Cq-3, *P. flexibilis* Cq-32, and *B. pumilus* Cq-35, however, these strains were statistically different from uninoculated control (**Figure 5D**). Strain *P. lucknowensis* Cq-48 reported maximum spike numbers with a 1.62-fold increase over uninoculated control (**Figure 6A**). This increase was statistically similar to the spike numbers due to strain *B. altitudinis* Cq-3 which showed a 1.49-fold increase compared to uninoculated control. The maximum increase in spike length was obtained due to inoculation with strains *P. lucknowensis* Cq-48 and *P. furukawaii* Cq-40, having a rise of 2.30 and 2.24-fold over uninoculated control (**Figure 6B**). These strains *P. lucknowensis* Cq-48 and *P. furukawaii* Cq-40, were statistically similar; however, they remain significant compared to uninoculated control. The spike numbers and spike length remain lowest in the uninoculated control treatment.

## Discussion

Eighteen rhizobacterial strains were characterized under insoluble minerals compounds, including P, Zn, and Mn. The tested rhizobacterial strains demonstrated remarkable solubilization of insoluble phosphate. Strains Cq-51 and Cq-40 reported maximum phosphate-solubilization zone diameter. However, maximum phosphate-solubilization index and efficiency were reported by strains Cq-40 and Cq-32. The maximum phosphate-solubilized concentration in the Pikovskaya medium amended with tri-calcium phosphate was observed due to strain Cq-48. These results are per those of Suleman et al. (2018); Wei et al. (2018); Baghel et al. (2020); Kour et al. (2020a); Liang et al. (2020); Pastore et al. (2020); Wang et al. (2021); Zhan et al. (2021), and Sahu et al. (2022). Maharana and Dhal (2022) recently isolated thirteen phosphates solubilizing bacterial (PSB) strains and found *Bacillus cereus* S_0_B_4_, *Solibacillus isronensis* S_0_B_8_, and *Bacillus amyloliquefaciens* S_0_B_17_ were the best solubilizers of rock-phosphate. *B*. *cereus* S_0_B_4_ corroded the rock-phosphate surface by producing protons (Maharana and Dhal, 2022). Li et al. (2022) reported a similar range of phosphate-solubilization zone diameter and phosphate-solubilization concentration along with the production of alkaline phosphatase enzyme. The phosphate-solubilization by these rhizobacterial strains might also be due to the production of organic and inorganic acids, siderophores, exopolysaccharides, hydrogen cyanide, and proton release or from ammonia assimilation (Rawat et al., 2021). In the current study, most tested PSB strains showed the production of malic, gluconic, ascorbic, lactic, and oxalic acids. Malic acid was produced by all the bacterial strains except Cq-3 and Cq-51. All the PSB strains had gluconic acid with maximum production by strain Cq-48. Tartaric acid was only produced by strain Cq-48. Half of the tested strains showed the production of ascorbic, lactic, and oxalic acids. Previously, phosphate-solubilization was reported due to the production of organic acids, including oxalic, malic, formic, acetic, tartaric, gluconic, lactic, fumaric, ascorbic, isocitric, citric, phytic, succinic, acetic, oxalic, propionic, glucuronic, butyric, valeric and isovaleric acids which dissolve insoluble source of phosphate in the amended Pikovskaya culture medium (Scervino et al., 2010; do Carmo et al., 2019; Bononi et al., 2020; Chawngthu et al., 2020; Rfaki et al., 2020). Rhizospheric bacterial strains adopted these mechanisms of action directly or indirectly to assimilate higher P concentrations from the soil through dissolving insoluble P compounds (Halvorson et al., 1990). Hegyi et al. (2021) reported the abundance of the alkaline phosphatase gene *phoD* in genera of proteobacteria involved in phosphate-solubilization, which might be valid for the current study.

In the present study, isolated PSB strains showed their potential in Zn solubilization. Strains Cq-40, Cq-47, Cq-51, and Cq-53 were better among tested PSB strains and demonstrated a more prominent solubilization zone diameter. Moreover, strain Cq-51 reported a maximum zinc solubilization index and zinc solubilization efficiency. Solubilization of Zn by PSB strains might accomplish by various mechanisms, including organic acids, proton extrusion, or chelating agents (Mumtaz et al., 2019; Zaheer et al., 2019; Kumar et al., 2021; Masood et al., 2022). The solubilization zone diameter may develop due to tested bacterial strains through media acidification. Similarly, Mumtaz et al. (2017) reported a direct relationship between the drop in pH of the medium and the increase in zinc availability. Our findings agree with earlier reports, where maximum Zn solubilization was achieved in the ZnO amended medium (Mumtaz et al., 2017; Khanghahi et al., 2018; Mumtaz et al., 2019; Zaheer et al., 2019; Bhatt and Maheshwari, 2020; Naseer et al., 2020; Bhakat et al., 2021; Karnwal, 2021; Naseem et al., 2022). Mumtaz et al. (2019) reported the production of lactic and acetic acids as primary Zn solubilizing acids along with citric, succinic, formic, isovaleric, and isobutyric acids as minor metabolic products produced by *Bacillus* strains in ZnO amended medium. Similarly, Zaheer et al. (2019) reported the ability of *Bacillus* and *Pseudomonas* strains to solubilize Zn related to the production of acetic, oxalic, gluconic, citric, lactic, and succinic acids. As suggested by a long list of potential acids, it may be feasible that any acid could solubilize Zn. Other acids, including gluconic, 5-ketogluconic, tartaric, and malic acids, may also be produced during the Zn solubilization assay, as reported by Fasim et al. (2002); Saravanan et al. (2007), and Dinesh et al. (2018).

The MSB strains were well-capable to solubilize Mn on nutrient agar medium amended with insoluble MnO_2_. Strains Cq-32 and Cq-31 reported a maximum Mn-solubilization zone. Maximum Mn-solubilization index and manganese solubilization efficiency was recorded from strain Cq-45. Previously, various researchers also reported Mn solubilization by microbial strains (Madgwick, 1991; Baglin et al., 1992; Wei et al., 2012; Mohanty et al., 2017; Sanket et al., 2017; Ghosh et al., 2018; Ghosh et al., 2021; Ijaz et al., 2021). Microbial strains including *Achromobacter* sp., *Aspergillus niger*, *Bacillus cereus* AMSB3, *Bacillus nealsonii* AMSB4, *Bacillus* sp. strains ASH6, ASH11, ASH19, ASH20, and ASH22, *Enterobacter agglomerans*, *Enterobacter cloacae*, *Enterobacter* sp. AMSB1 and *Staphylococcus hominis* AMSB5 were previously demonstrated to solubilize insoluble Mn (Madgwick, 1991; Sanket et al., 2017; Ijaz et al., 2021). Mn solubilization in the current study may be due to the ability of bacterial strains to reduce Mn(IV) and Mn(III) into Mn(II). They reduced Mn(IV) enzymatically and oxidized Mn(II), which served as a terminal electron acceptor for anaerobic and facultative anaerobic bacteria, and consumed it to fulfill their nutritional needs (Bromfield and David, 1976).

The selected PSB strains were identified as *B. altitudinis* Cq-3, *P. flexibilis* Cq-32, *B. pumilus* Cq-35, *P. furukawaii* Cq-40, *P*. *lucknowensis* Cq-48, and *Ensifer* sp. Cq-51 through 16S rRNA gene sequencing. These strains showed indole acetic acid production both in the presence and absence of L-tryptophan. However, indole acetic acid production was higher in the presence of L-tryptophan as it is a highly efficient physiological precursor of auxins. In the absence of L-tryptophan, strains P. lucknowensis Cq-48 and Ensifer sp observed maximum indole acetic acid production. Cq-51. Increasing IAA production by MSB strains may promote primary root elongation and the formation of lateral and adventitious roots (Xie et al., 1996). Previously *in vitro* studies showed that bacterial strains could produce a small concentration of auxins in the absence of L-tryptophan; however, in its presence, the bacterial strains showed a remarkable increase in auxin production (Zahir et al., 2010). Panigrahi et al. (2020) reported variation in the production of IAA by endophytic *E*. *cloacae* MG00145 isolated from *Ocimum sanctum* and significantly promoted the growth of various crops. Soil treatment with exogenous L-tryptophan also positively affected the synthesis of auxins and plant growth (Khalid et al., 2004). The IAA production in the absence of L-tryptophan might improve root growth in terms of root length and dry weight of quinoa in the present pot and field trials. The root is the main organ absorbing minerals and water and participates in material transport (Wang et al., 2006). Therefore, enhanced IAA production is involved in the promotion of fresh and dry plant weight and plant height by increasing the root system.

In the current pot and field trials, the inoculation of quinoa seeds with MSB strains significantly promoted growth attributes, including plant height, leaves count, root length, shoot and root dry weight. Quinoa seed coated with strains *P*. *lucknowensis* Cq-48 followed by *Ensifer* sp. Cq-51, *B. altitudinis* Cq-3, and *P. flexibilis* Cq-32 showed the highest increase in these attributes. More fabulous foliage and dry biomass may be linked to increases in the number of leaves, plant height, and root length that improve the minerals uptake and enhanced photosynthetic rates compared to uninoculated control. In the current study, seed-applied bacterial strains of the genus *Bacillus*, *Pseudomonas*, *Pontibacter*, and *Ensifer* encourage plant growth that might be due to the production of growth hormones, including IAA that might promote cell division and enlargement. IAA is also helpful in tissue expansion that causes an increase in plant height and root length, and biomass accumulation. It promoted root hair formation by regulating root elongation and the development of lateral roots (Naqqash et al., 2016). The increased surface area of the root in terms of root hair also promoted nutrient uptake efficiency of the plant and resulted in better plant growth. Several other growth hormones, including cytokinin, gibberellic acid, and salicylic acid production by bacterial strains, which were not studied in the current study, are also involved stimulation of cell division and tissue expansion that ultimately promote plant height, root length, and biomass accumulation (Ekinci et al., 2014; Ruzzi and Aroca, 2015).

This study found significantly higher relative humidity levels, quantum flux, diffusive resistance, and transpiration rate in MSB strains in inoculated plants than in uninoculated ones. The increase in these physiological attributes could be due to the positive effect of MSB strains tested in the current study. The strain *P*. *lucknowensis* Cq-48 followed by *P. flexibilis* Cq-32, and *P. furukawaii* Cq-40 reported maximum values for relative humidity, quantum flux, diffusive resistance, and transpiration rate. These strains showed an increase in quantum flux and transpiration rate, representing an increase in chlorophyll contents and photosynthetic rates. Previously, strains of the genus *Pseudomonas*, *Bacillus*, *Pontibacter*, and *Ensifer* spp. increased chlorophyll contents, photosynthesis, and transpiration rate (Chu et al., 2006; Bulegon et al., 2016; Chakraborty et al., 2019; Saha et al., 2021). The increase in chlorophyll contents as a result of enhanced quantum flux, transpiration rate, and leave numbers, as found in the current study, promotes photosynthetic activity, which leads to improved growth and yield of inoculated quinoa plants. The maximum quantum flux of photosystem-II is a crucial indicator of the photosynthetic performance of plants (Vishnupradeep et al., 2022). Bacterial inoculation protects the photosystem-II reaction center and regulates the photosynthetic electron transport by reducing the stress condition regarding nutrient deficiencies and biotic and abiotic stresses (Win et al., 2018). Bacterial strains also held antioxidant activities, relative humidity, secondary metabolites accumulation, CO_2_ fixation, and improved net photosynthesis, which resulted in the increased growth rate of plants (Taj and Challabathula, 2021).

In this study, the increase in growth and yield attributes could be due to the rise in minerals uptake due to inoculation with minerals-solubilizing bacterial strains that can solubilize P, Zn, and Mn. The bacterial strains enhanced nutrient availability would significantly promote plant growth under nutrient-depleted conditions (Swarnalakshmi et al., 2020). In the current study, enhanced minerals uptake encourages the availability of nitrogen which enhance vegetative plant growth, including length and biomass of roots, shoots, and number of leaves. We do not test nitrogen fixation by the bacterial strains, however, tested bacterial strains of the genus *Bacillus*, *Pseudomonas*, *Pontibacter*, and *Ensifer* were previously reported for nitrogen fixation and their role in the promotion of vegetative growth (Liu et al., 2019; Sibponkrung et al., 2020; diCenzo et al., 2021; Geries and Elsadany, 2021). Our tested strains were efficient P solubilizers that may promote P uptake by plants and play their role in cellular division and tissue formation (Perez-Torres et al., 2008; Yu et al., 2016). The tested strains were good organic acid producers which solubilize most of the fixed/precipitated minerals, including potassium. Potassium availability is vital during the early fruit stage of producing seeds, which might be valid for the current study through bacterial solubilization of potassium. de Sousa et al. (2021) reported that maize inoculated with *Bacillus* strains having the ability to solubilize P and IAA production enhanced root morphology, dry matter, nutrient accumulation, and higher yield. Similarly, Adhikari et al. (2021) reported the plant growth promotion of *Arabidopsis thaliana* plants through inoculation with *Pseudomonas palleroniana* GBPI-508. The presence of multi-traits characteristics in all the tested MSB strains could provide an opportunity for formulating biofertilizers which could be helpful in the bioavailability of P, Zn, and Mn, IAA production, production of organic acids, and promotion of quinoa plant growth, physiology, and yield attributes.

## Conclusion

The present study concluded that *Bacillus altitudinis* Cq-3, *Pseudomonas flexibilis* Cq-32, *Bacillus pumilus* Cq-35, *Pseudomonas furukawaii* Cq-40, *Pontibacter lucknowensis* Cq-48, *and Ensifer* sp. Cq-51 showed strong power to solubilize phosphate, zinc, and manganese. These strains showed the production of organic acids in tri-calcium amended medium and indole-3-acetic output in the presence and absence of L-tryptophan. These strains also improved quinoa growth attributes (plant height, root length, spike length, leaves, spikes count, shoot and root dry weight) and physiological attributes (relative humidity, quantum flux, diffusive resistance, and transpiration rate) in the pot and field conditions. *Pontibacter lucknowensis* Cq-48 followed by *Pseudomonas furukawaii* Cq-40 and *Pseudomonas flexibilis* Cq-32 showed promising results and significantly promoted quinoa growth and physiology. Such prospective bioinoculants could address the issue of mineral deficiencies in wild plants, especially quinoa. The present study suggests researchers evaluate these selected strains to study their genetic and molecular mechanisms for minerals solubilization and plant growth promotion in nutrient-deficient conditions.

## Data availability statement

The data presented in the study are deposited in the DNA data bank of Japan (DDBJ) repository: Bacillus altitudinis Cq-3 accession number LC667775 (https://www.ncbi.nlm.nih.gov/nuccore/LC667775), Pseudomonas flexibilis Cq32 accession number LC667802 https://www.ncbi.nlm.nih.gov/nuccore/LC667802), Bacillus pumilus Cq-35 accession number LC667805 (https://www.ncbi.nlm.nih.gov/nuccore/LC667805), Pseudomonas furukawaii Cq-40 accession number LC667809 (https://www.ncbi.nlm.nih.gov/nuccore/LC667809), Pontibacter lucknowensis Cq-48 accession number LC667816 (https://www.ncbi.nlm.nih.gov/nuccore/LC667816), Ensifer sp. Cq-51 accession number LC667819 (https://www.ncbi.nlm.nih.gov/nuccore/LC667819).

## Author contributions

ER and MM conceived and designed the experimental strategies. ER performed all experiments and data analysis. MM provided study materials, reagents, and research resources and facilitated manuscript preparation. ER, KQ and MM created and presented the published work, explicitly writing the initial and final draft and performing revision. IU helped in the identification of bacterial strains and performed phylogenetic analysis. IU and MJ oversight the research activity, planning, and execution. KQ, MM, MK, and MJ did formal analysis of the data. MK, MJ, KQ, AR, and MR critically review the manuscript and helped with manuscript revision. MJ, KQ, and MA provided financial support for the publication of this article. All authors contributed to the article and approved the submitted version.

## Funding

The APC was funded by the King Abdullah University of Science and Technology (KAUST), Thuwal, Saudi Arabia.

## Acknowledgments

The current study is a part of the Ph.D. dissertation of Mr. Ejaz Rafique (the first author) and he would like to express his gratitude to the Microbial Biotechnology Laboratory, Institute of Molecular Biology and Biotechnology (IMBB), The University of Lahore (UOL), Pakistan, for providing the working space and research facilities. The authors highly acknowledged the help from Dr. Ahmad Zaheer (Ex. Assistant Professor, IMBB, UOL, Lahore, Pakistan) during the methodology of this work. The authors also thank Prof. Dr. Muhammad Yaseen Ashraf (IMBB, UOL, Lahore, Pakistan) for facilitating the leaf porometer. The authors would like to express their deepest gratitude to University of Tabuk, for the technical support for this study.

## Conflict of interest

The authors declare that the research was conducted in the absence of any commercial or financial relationships that could be construed as a potential conflict of interest.

## Publisher’s note

All claims expressed in this article are solely those of the authors and do not necessarily represent those of their affiliated organizations, or those of the publisher, the editors and the reviewers. Any product that may be evaluated in this article, or claim that may be made by its manufacturer, is not guaranteed or endorsed by the publisher.

## References

[B1] Adhikari P. Jain R. Sharma A. Pandey A. (2021). Plant growth promotion at low temperature by phosphate-solubilizing *Pseudomonas* spp. isolated from high-altitude himalayan soil. Microbial. Ecol. 82 (3), 677–687. doi: 10.1007/s00248-021-01702-1 33512536

[B2] Al-Barakah F. N. Sohaib M.. (2019). Evaluating the germination response of Chenopodium quinoa seeds to bacterial inoculation under different germination media and salinity conditions. Seed Science and Technology 47 (2), 161–9. doi: 10.15258/sst.2019.47.2.05

[B3] Alori E. T. Babalola O. O. (2018). Microbial inoculants for improving crop quality and human health in Africa. Front. Microbiol. 9. doi: 10.3389/fmicb.2018.02213 PMC6156547 30283427

[B4] Alori E. T. Fawole O. B. (2017). “Microbial inoculants-assisted phytoremediation for sustainable soil management,” in Phytoremediation: Management of environmental contaminants, Switzerland. Eds. Ansari A. A. Gill S. S. Lanza G. R. Newman L. (Berlin: Springer International Publishing). doi: 10.1007/978-3-319-52381-1_1

[B5] Amy C. Avice J. C. Laval K. Bressan M. (2022). Are native phosphate solubilizing bacteria a relevant alternative to mineral fertilizations for crops? part i. when rhizobacteria meet plant p requirements. Rhizosphere 21, 100476. doi: 10.1016/j.rhisph.2022.100476

[B6] Baghel V. Thakur J. K. Yadav S. S. Manna M. C. Mandal A. Shirale A. O.. (2020). Phosphorus and potassium solubilization from rock minerals by endophytic *Burkholderia* sp. strain FDN2-1 in soil and shift in diversity of bacterial endophytes of corn root tissue with crop growth stage. Geomicrobiol. J. 37 (6), 550–563. doi: 10.1080/01490451.2020.1734691

[B7] Baglin E. G. Noble E. G. Lamsphire D. L. Eisele J. A. (1992). Solubilization of manganese from ores by heterotrophic micro-organisms. Hydrometallurgy 29 (1-3), 131–144. doi: 10.1016/0304-386X(92)90009-O

[B8] Bhakat K. Chakraborty A. Islam E. (2021). Characterization of zinc solubilization potential of arsenic tolerant *Burkholderia* spp. isolated from rice rhizospheric soil. World J. Microbiol. Biotech. 37 (3), 1–13.doi: 10.1007/s11274-021-03003-8 33544268

[B9] Bhatt K. Maheshwari D. K. (2020). Zinc solubilizing bacteria (*Bacillus megaterium*) with multifarious plant growth promoting activities alleviates growth in *Capsicum annuum* l. 3 Biotech. 10 (2), 1–10. doi: 10.1007/s13205-019-2033-9 31988830 PMC6946769

[B10] Bononi L. Chiaramonte J. B. Pansa C. C. Moitinho M. A. Melo I. S. (2020). Phosphorus-solubilizing *Trichoderma* spp. from Amazon soils improve soybean plant growth. Sci. Rep. 10, 1–13. doi: 10.1038/s41598-020-59793-8 32071331 PMC7028723

[B11] Bromfield S. M. David D. J. (1976). Sorption and oxidation of manganous ions and reduction of manganese oxide by cell suspensions of a manganese oxidizing bacterium. Soil Biol. Biochem. 8, 37–43. doi: 10.1016/0038-0717(76)90019-5

[B12] Bulegon L. G. Guimarães V. F. Laureth J. C. U. (2016). *Azospirillum brasilense* affects the antioxidant activity and leaf pigment content of urochloa ruziziensis under water stress. Pesqui. Agropecuária. Trop. 46, 343–349. doi: 10.1590/1983-40632016v4641489

[B13] Cauda C. Micheletti C. Minerdo B. Scaffidi C. Signoroni E. (2013). Quinoa in the kitchen (Rome: Food and Agricultural organization of United Nations (FAO).

[B14] Chakraborty U. Roy S. Chakraborty B. (2019). Microorganisms aiding existence and efficiency of plants in saline environment: what we know and what to expect. in microorganisms in saline environments: Strategies and functions (Cham: Springer), (pp. 211–235).

[B15] Chawngthu L. Hnamte R. Lalfakzuala R. Geomicrobiol. J. (2020). Isolation and characterization of rhizospheric phosphate solubilizing bacteria from wetland paddy field of mizoram, India Geomicrobiology Journal 37, 366–375. doi: 10.1080/01490451.2019.1709108

[B16] Chumpitaz-Segovia C. Alvarado D. Ogata-Gutiérrez K. Zúñiga-Dávila D. (2020). Bioprospection of native psychrotolerant plant-growth-promoting rhizobacteria from Peruvian Andean plateau soils associated with *Chenopodium quinoa* . Canad. J. Microbiol. 66 (11), 641–652. doi: 10.1139/cjm-2020-0036 32574514

[B17] Chu W. Zhengquan W. Hailong S. Shenglei G. (2006). Effects of different concentrations of nitrogen and phosphorus on chlorophyll biosynthesis, chlorophyll a fluorescence, and photosynthesis in *Larix olgensis* seedlings. Front. For. China 1, 170–175. doi: 10.1007/s11461-006-0019-3

[B18] de Sousa S. M. de Oliveira C. A. Andrade D. L. de Carvalho C. G. Ribeiro V. P. Pastina M. M.. (2021). Tropical *Bacillus* strains inoculation enhances maize root surface area, dry weight, nutrient uptake and grain yield. J. Plant Growth Regul. 40 (2), 867–877. doi: 10.1007/s00344-020-10146-9

[B19] diCenzo G. C. Cangioli L. Nicoud Q. Cheng J. H. Blow M. J. Shapiro N.. (2021). DNA Methylation in *Ensifer* species during free-living growth and during nitrogen-fixing symbiosis with *Medicago* spp. mSystems 7, 1, e01092–e01021. doi: 10.1128/mSystems.01092-21 PMC8725594 35089065

[B20] Dinesh R. Srinivasan V. Hamza S. Sarathambal C. Gowda S. J. A. Ganeshamurthy A. N.. (2018). Isolation and characterization of potential zn solubilizing bacteria from soil and its effects on soil zn release rates, soil available zn and plant zn content. Geoderma 321, 173–186. doi: 10.1016/j.geoderma.2018.02.013

[B21] Divjot K. O. U. R. Rana K. L. Tanvir K. A. U. R. Yadav N. Yadav A. N. Kumar M.. (2021). Biodiversity, current developments and potential biotechnological applications of phosphorus-solubilizing and-mobilizing microbes: a review. Pedosphere 31 (1), 43–75. doi: 10.1016/S1002-0160(20)60057-1

[B22] do Carmo T. S. Moreira F. S. Cabral B. V. Dantas R. C. de Resende M. M. Cardoso V. L.. (2019). Phosphorus recovery from phosphate rocks using phosphate-solubilizing bacteria. Geomicrobiol. J. 36, 195–203. doi: 10.1080/01490451.2018.1534901

[B23] Ekinci M. Turan M. Yildirim E. Güne A. Kotan R. Dursun A. (2014). Effect of plant growth promoting rhizobacteria on growth, nutrient, organic acid, amino acid and hormone content of cauliflower (Brassica oleracea l. var. botrytis) transplants. Acta Sci. Pol. Hortic. 13, 71–85.

[B24] Fasim F. Ahmed N. Parsons R. Gadd G. M. (2002). Solubilization of zinc salts by a bacterium isolated from air environment of a tannery. FEMS Microbiol. Lett. 213, 1–6. doi: 10.1111/j.1574-6968.2002.tb11277.x 12127480

[B25] Geries L. S. M. Elsadany A. Y. (2021). Maximizing growth and productivity of onion (Allium cepa l.) by *Spirulina platensis* extract and nitrogen-fixing endophyte *Pseudomonas stutzeri* . Arch. Microbiol. 203 (1), 169–181. doi: 10.1007/s00203-020-01991-z 32789754

[B26] Ghosh S. Bal B. Das A. P. (2018). Enhancing manganese recovery from low-grade ores by using mixed culture of indigenously isolated bacterial strains. Geomicrobiol. J. 35 (3), 242–246. doi: 10.1080/01490451.2017.1362080

[B27] Ghosh S. Gandhi M. van Hullebusch E. D. Das A. P. (2021). Proteomic insights into *Lysinibacillus* sp.-mediated biosolubilization of manganese. Environ. Sci. pollut. Res. 28 (30), 40249–40263. doi: 10.1007/s11356-020-10863-4 33011949

[B28] Guo H. Hao Y. Yang X. Ren G. Richel A. (2021). Exploration on bioactive properties of quinoa protein hydrolysate and peptides: a review. Crit. Rev. Food Sci. Nutr. 28, 1–14. doi: 10.1080/10408398.2021.1982860 34581209

[B29] Halvorson H. O. Keynan A. Kornberg H. L. (1990). Utilization of calcium phosphates for microbial growth at alkaline pH. Soil Biol. Biochem. 22, 887–890. doi: 10.1016/0038-0717(90)90125-J

[B30] Hariprasad P. Niranjana S. (2009). Isolation and characterization of phosphate solubilizing rhizobacteria to improve plant health of tomato. Plant Soil 316, 13–24. doi: 10.1007/s11104-008-9754-6

[B31] Hegyi A. Nguyen T. B. K. Posta K. (2021). Metagenomic analysis of bacterial communities in agricultural soils from Vietnam with special attention to phosphate solubilizing bacteria. Microorganisms 9 (9), 1796. doi: 10.3390/microorganisms9091796 34576692 PMC8472641

[B32] Ijaz A. Mumtaz M. Z. Wang X. Ahmad M. Saqib M. Maqbool H.. (2021). Insights into manganese solubilizing bacillus spp. for improving plant growth and manganese uptake in maize. Front. Plant Sci. 12. doi: 10.3389/fpls.2021.719504 PMC8593242 34795682

[B33] Jacobsen S. E. (2003). The worldwide potential for quinoa (*Chenopodium quinoa* willd.). Food Rev. Int. 19 (1-2), 167–177. doi: 10.1081/FRI-120018883

[B34] Jaikishun S. Li W. Yang Z. Song S. (2019). Quinoa: In perspective of global challenges. Agronomy 9 (4), 176. doi: 10.3390/agronomy9040176

[B35] Karnwal A. (2021). Zinc solubilizing pseudomonas spp. from vermicompost bestowed with multifaceted plant growth promoting properties and having prospective modulation of zinc biofortification in *Abelmoschus esculentus* l. J. Plant Nutr. 44 (7), 1023–1038. doi: 10.1080/01904167.2020.1862199

[B36] Katsivela E. Bonse D. Krüger A. Strömpl C. Livingston A. Wittich R. M. (1999). An extractive membrane biofilm reactor for degradation of 1, 3-dichloropropene in industrial waste water. Appl. Microbiol. Biotech. 52 (6), 853–862. doi: 10.1007/s002530051603 10616720

[B37] Khalid M. Hassani D. Bilal M. Asad F. Huang D. (2017). Influence of bio-fertilizer containing beneficial fungi and rhizospheric bacteria on health promoting compounds and antioxidant activity of *Spinacia oleracea* l. Bot. Stud. 58, 35. doi: 10.1186/s40529-017-0189-3 28815474 PMC5559411

[B38] Khalid A. Tahir S. Arshad M. Zahir Z. A. (2004). Relative efficiency of rhizobacteria for auxin biosynthesis in rhizosphere and nonrhizosphere soils. Aust. J. Soil Res. 42, 921–926. doi: 10.1071/SR04019

[B39] Khanghahi M. Y. Ricciuti P. Allegretta I. Terzano R. Crecchio C. (2018). Solubilization of insoluble zinc compounds by zinc solubilizing bacteria (ZSB) and optimization of their growth conditions. Environ. Sci. Pol. Res. 25 (26), 25862–25868. doi: 10.1007/s11356-018-2638-2 29959742

[B40] Kour D. Rana K. L. Kaur T. Sheikh I. Yadav A. N. Kumar V.. (2020a). Microbe-mediated alleviation of drought stress and acquisition of phosphorus in great millet (*Sorghum bicolour* l.) by drought-adaptive and phosphorus-solubilizing microbes. Biocatal. Agric. Biotechnol. 23, 101501. doi: 10.1016/j.bcab.2020.101501

[B41] Kour D. Rana K. L. Yadav A. N. Yadav N. Kumar M. Kumar V.. (2020b). Microbial biofertilizers: Bioresources and eco-friendly technologies for agricultural and environmental sustainability. Biocatal. Agric. Biotechnol. 23, 101487. doi: 10.1016/j.bcab.2019.101487

[B42] Kumar U. Malik R. Malik K. (2021). “Fe chelation and zinc solubilization: A promising approach for cereals biofortification,” in Soil microbiomes for sustainable agriculture (Cham: Springer), 149–174.

[B43] Kumar V. Singh P. Jorquera M. A. Sangwan P. Kumar P. Verma A.. (2013). Isolation of phytase-producing bacteria from Himalayan soils and their effect on growth and phosphorus uptake of Indian mustard (*Brassica juncea*). World J. Microbol. Biotechnol. 29, 1361–1369. doi: 10.1007/s11274-013-1299-z 23546828

[B44] Laghari K. A. Sial M. A. Arain M. A. Mirbahar A. A. Pirzada A. Dahot M.. (2010). Heritability studies of yield and yield associated traits in bread wheat. Pak. J. Bot. 42 (1), 111–115.

[B45] Lalitha S. (2017). “Plant growth–promoting microbes: a boon for sustainable agriculture,” in Sustainable agriculture towards food security. Ed. Dhanarajan A. (Singapore: Springer Singapore), 125–158. doi: 10.1007/978-981-10-6647-4_8

[B46] Leite M.C.D.B.S. Pereira A. P. D. A. Souza A. J. D. Andreote F. D. Freire F. J. Sobral J. K. (2018). Bioprospection and genetic diversity of endophytic bacteria associated with cassava plant. Rev. Caatinga. 31, 315–325. doi: 10.1590/1983-21252018v31n207rc

[B47] Liang J. L. Liu J. Jia P. Yang T. T. Zeng Q. W. Zhang S. C.. (2020). Novel phosphate-solubilizing bacteria enhance soil phosphorus cycling following ecological restoration of land degraded by mining. ISME. J. 14 (6), 1600–1613. doi: 10.1038/s41396-020-0632-4 32203124 PMC7242446

[B48] Liu Z. W. Zhang J. Yu Y. Li H. R. Du Z. J. (2019). *Pontibacter arcticus* sp. nov., isolated from rhizosphere soil of *Saxifraga oppositifolia* . Int. J. Syst. Evol. Microbiol. 69 (11), 3609–3615. doi: 10.1099/ijsem.0.003668 31436526

[B49] Li Y. Yu X. Zheng J. Gong Z. Xu W. (2022). Diversity and phosphate solubilizing characteristics of cultivable organophosphorus-mineralizing bacteria in the sediments of sancha lake. Int. J. Environ. Res. 19 (4), 2320. doi: 10.3390/ijerph19042320 PMC8872205 35206506

[B50] Mącik M. Gryta A. Frąc M. (2020). Biofertilizers in agriculture: An overview on concepts, strategies and effects on soil microorganisms. Adv. Agron. 162, 31–87. doi: 10.1016/bs.agron.2020.02.001

[B51] Madgwick J. C. (1991). Microbial processing of manganese. Aust. J. Biotechnol. 12, 33–38.

[B52] Maharana R. Dhal N. K. (2022). Solubilization of rock phosphate by phosphate solubilizing bacteria isolated from effluent treatment plant sludge of a fertilizer plant. Folia Microbiol. 11, 605–615. doi: 10.1007/s12223-022-00953-w 35277802

[B53] Mahdi I. Fahsi N. Hafidi M. Allaoui A. Biskri L. (2020). Plant growth enhancement using rhizospheric halotolerant phosphate solubilizing bacterium *Bacillus licheniformis* QA1 and *Enterobacter asburiae* QF11 isolated from *Chenopodium quinoa* willd. Microorganisms 8 (6), 948. doi: 10.3390/microorganisms8060948 PMC7356859 32599701

[B54] Maliro M. F. Abang M. M. Mukankusi C. Lung’aho M. Fenta B. Wanderi S.. (2021). Prospects for quinoa adaptation and utilization in Eastern and southern Africa: Technological, institutional and policy considerations. Food Agric. Org. doi: 10.4060/cb2351en

[B55] Martínez-Gallegos V. Bautista-Cruz A. Martínez-Martínez L. Medina S. S. P. (2018). First report of phosphate-solubilizing bacteria associated with agave angustifolia. Int. J. Agric. Biol. 20, 1298–1302. doi: 10.17957/IJAB/15.0630

[B56] Masood F. Ahmad S. Malik A. (2022). Role of rhizobacterial bacilli in zinc solubilization. in microbial biofertilizers and micronutrient availability (Cham: Springer), 361–377.

[B57] Mishra P. K. Joshi P. Suyal P. Bisht J. K. Bhatt J. C. (2014). “Potential of phosphate solubilising microorganisms in crop production,” in Bioresources for sustainable plant nutrient management (New Delhi, India: Satish Serial Publishing House), 201–222.

[B58] Mohanty S. Ghosh S. Nayak S. Das A. P. (2017). Isolation, identification, and screening of manganese solubilizing fungi from low-grade manganese ore deposits. Geomicrobiol. J. 34 (4), 309–316. doi: 10.1080/01490451.2016.1189016

[B59] Mumtaz M. Z. Ahmad M. Jamil M. Hussain T. (2017). Zinc solubilizing *Bacillus* spp. potential candidates for biofortification in maize. Microbiol. Res. 202, 51–60. doi: 10.1016/j.micres.2017.06.001 28647123

[B60] Mumtaz M. Z. Ahmad M. Zafar-ul-Hye M. Saqib M. Akhtar M. F. U. Z. Zaheer M. S.. (2022). Seed-applied zinc-solubilising *Bacillus* biofertilisers improve antioxidant enzyme activities, crop productivity, and biofortification of maize. Crop Pasture Sci 73 (5), 503–514. doi: 10.1071/CP21415

[B61] Mumtaz M. Z. Barry K. M. Baker A. L. Nichols D. S. Ahmad M. Zahir Z. A.. (2019). Production of lactic and acetic acids by bacillus sp. ZM20 and *Bacillus cereus* following exposure to zinc oxide: A possible mechanism for zn solubilization. Rhizosphere 12, 100170. doi: 10.1016/j.rhisph.2019.100170

[B62] Naqqash T. Hameed S. Imran A. Hanif M. K. Majeed A. van Elsas J. D. (2016). Differential response of potato toward inoculation with taxonomically diverse plant growth promoting rhizobacteria. Front. Plant Sci. 7. doi: 10.3389/fpls.2016.00144 PMC4756182 26925072

[B63] Naseem S. Hussain A. Wang X. Iqbal Z. Mustafa A. Mumtaz M. Z.. (2022). Exopolysaccharide and siderophore production ability of zn solubilizing bacterial strains improve growth, physiology and antioxidant status of maize and wheat. Pol. J. Environ. Stud. 31 (2), 1223–1236. doi: 10.15244/pjoes/140563

[B64] Naseer I. Ahmad M. Hussain A. Jamil M. (2020). Potential of zinc solubilizing *Bacillus* strains to improve rice growth under axenic conditions. Pak. J. Agric. Sci. 57, 1057–1071. doi: 10.21162/PAKJAS/20.9988

[B65] Navruz-Varli S. Sanlier N. (2016). Nutritional and health benefits of quinoa (*Chenopodium quinoa* willd.). J. Cereal Sci. 69, 371–376. doi: 10.1016/j.jcs.2016.05.004

[B66] Panigrahi S. Mohanty S. Rath C. C. (2020). Characterization of endophytic bacteria *Enterobacter cloacae* MG00145 isolated from *Ocimum sanctum* with indole acetic acid (IAA) production and plant growth promoting capabilities against selected crops. South Afr. J. Bot. 134, 17–26. doi: 10.1016/j.sajb.2019.09.017

[B67] Park K. H. Lee O. M. Jung H. I. Jeong J. H. Jeon Y. D. Hwang D. Y.. (2010). Rapid solubilization of insoluble phosphate by a novel environmental stress-tolerant *Burkholderia vietnamiensis* M6 isolated from ginseng rhizospheric soil. Appl. Microbiol. Biotech. 86 (3), 947–955. doi: 10.1007/s00253-009-2388-7 20024543

[B68] Pastore G. Kernchen S. Spohn M. (2020). Microbial solubilization of silicon and phosphorus from bedrock in relation to abundance of phosphorus-solubilizing bacteria in temperate forest soils. Soil Biol. Bioch. 151, 108050. doi: 10.1016/j.soilbio.2020.108050

[B69] Penn C. J. Camberato J. J. (2019). A critical review on soil chemical processes that control how soil pH affects phosphorus availability to plants. Agriculture 9 (6), 120. doi: 10.3390/agriculture9060120

[B70] Perez-Torres C. A. Lopez-Bucio J. Cruz-Ramırez A. Ibarra-Laclette E. Dharmasiri S. Estelle M.. (2008). Phosphate availability alters lateral root development in arabidopsis by modulating auxin sensitivity *via* a mechanism involving the tir1 auxin receptor. Plant Cell 20, 3258–3272. doi: 10.1105/tpc.108.058719 19106375 PMC2630440

[B71] Pikovskaya R. (1948). Mobilization of phosphorus in soil in connection with vital activity of some microbial species. Mikrobiologiya 17, 362–370.

[B72] Rawat P. Das S. Shankhdhar D. Shankhdhar S. C. (2021). Phosphate-solubilizing microorganisms: mechanism and their role in phosphate solubilization and uptake. J. Soil Sci. Plant Nutr. 21 (1), 49–68. doi: 10.1007/s42729-020-00342-7

[B73] Rfaki A. Zennouhi O. Aliyat F. Z. Nassiri L. Ibijbijen J. (2020). Isolation, selection and characterization of root-associated rock phosphate solubilizing bacteria in moroccan wheat (*Triticum aestivum* l.). Geomicrobiol. J. 37, 230–241. doi: 10.1080/01490451.2019.1694106

[B74] Ruzzi M. Aroca R. (2015). Plant growth-promoting rhizobacteria act as biostimulants in horticulture. Sci. Hortic. 196, 124–134. doi: 10.1016/j.scienta.2015.08.042

[B75] Ryan J. G. Estefan G. Rashid A. (2001). Soil and plant analysis laboratory manual (Aleppo: International Center for Agricultural Research in the Dry Areas (ICARDA).

[B76] Saha L. Tiwari J. Bauddh K. Ma Y. (2021). Recent developments in microbe-plant-based bioremediation for tackling heavy metal-polluted soils. Front. Microb. 12. doi: 10.3389/fmicb.2021.731723 PMC8733405 35002995

[B77] Sahu S. Rajbonshi M. P. Gujre N. Gupta M. K. Shelke R. G. Ghose A.. (2022). Bacterial strains found in the soils of a municipal solid waste dumping site facilitated phosphate solubilization along with cadmium remediation. Chemosphere 287, 132320. doi: 10.1016/j.chemosphere.2021.132320 34826951

[B78] Sanket A. Ghosh S. Sahoo R. Nayak S. Das A. (2017). Molecular identification of acidophilic manganese (Mn)-solubilizing bacteria from mining effluents and their application in mineral beneficiation. Geomicrobiol. J. 34 (1), 71–80. doi: 10.1080/01490451.2016.1141340

[B79] Saravanan V. S. Kalaiarasan P. Madhaiyan M. Thangaraju M. (2007). Solubilization of insoluble zinc compounds by *Gluconacetobacter diazotrophicus* and the detrimental action of zinc ion (Zn^2+^) and zinc chelates on root knot nematode *Meloidogyne incognita* . Lett. Appl. Microbiol. 44, 235–241. doi: 10.1111/j.1472-765X.2006.02079.x 17309498

[B80] Scervino J. M. Mesa M. P. Della Monica I. Recchi M. Moreno N. S. Godeas A. (2010). Soil fungal isolates produce different organic acid patterns involved in phosphate salts solubilization. Biol. Fertil. Soils. 46, 755–763. doi: 10.1007/s00374-010-0482-8

[B81] Sibponkrung S. Kondo T. Tanaka K. Tittabutr P. Boonkerd N. Yoshida K. I.. (2020). Co-Inoculation of *Bacillus velezensis* strain S141 and *Bradyrhizobium* strains promotes nodule growth and nitrogen fixation. Microorganisms 8 (5), 678. doi: 10.3390/microorganisms8050678 PMC7284691 32392716

[B82] Singh R. Kumar A. Singh M. Pandey K. D. (2019). “Isolation and characterization of plant growth promoting rhizobacteria from momordica charantia l,” in PGPR amelioration in sustainable agriculture. Eds. Singh A. K. Kumar A. Singh P. K. (Woodhead Publishing), 217–238.

[B83] Soetan K. Olaiya C. Oyewole O. (2010). The importance of mineral elements for humans, domestic animals and plants-a review. Afr. J. Food Sci. 4, 200–222. doi: 10.5897/AJFS.9000287

[B84] Somasegaran P. Hoben H. J. (1994). Quantifying the growth of rhizobia. in handbook for rhizobia (New York: Springer), 47–57.

[B85] Steel R. Torrie J. Dicky D. (1997). Principles and procedures of statistics, a biometrical approach. 3rd Edn (New York: McGraw Hill, Inc. Book Co).

[B86] Suleman M. Yasmin S. Rasul M. Yahya M. Atta B. M. Mirza M. S. (2018). Phosphate solubilizing bacteria with glucose dehydrogenase gene for phosphorus uptake and beneficial effects on wheat. PloS One 13 (9), e0204408. doi: 10.1371/journal.pone.0204408 30240432 PMC6150522

[B87] Suyal D. C. Soni R. Sai S. Goel R. (2016). Microbial inoculants as biofertilizer, in microbial inoculants in sustainable agricultural productivity. Ed. Singh D. P. (New Delhi: Springer India), 311–318. doi: 10.1007/978-81-322-2647-5_18

[B88] Swarnalakshmi K. Yadav V. Tyagi D. Dhar D. W. Kannepalli A. Kumar S. (2020). Significance of plant growth promoting rhizobacteria in grain legumes: Growth promotion and crop production. Plants 9 (11), 1596. doi: 10.3390/plants9111596 PMC7698556 33213067

[B89] Taj Z. Challabathula D. (2021). Protection of photosynthesis by halotolerant staphylococcus sciuri ET101 in tomato (*Lycoperiscon esculentum*) and rice (*Oryza sativa*) plants during salinity stress: Possible interplay between carboxylation and oxygenation in stress mitigation. Front. Microbiol. 11, 547750. doi: 10.3389/fmicb.2020.547750 33488529 PMC7820118

[B90] Tamura K. Dudley J. Nei M. Kumar S. (2007). MEGA4: molecular evolutionary genetics analysis (MEGA) software version 4.0. Mol. Biol. Evol. 24 (8), 1596–1599. doi: 10.1093/molbev/msm092 17488738

[B91] Tan M. (2020). Macro-and micromineral contents of different quinoa (*Chenopodium quinoa* willd.) varieties used as forage by cattle. Turk. J. Agric. Forest. 44 (1), 46–53. doi: 10.3906/tar-1904-10

[B92] Testen A. L. Claros Magnus M. I. Backman P. A. (2022). Plant growth promoting traits of *Bacillus* species associated with quinoa (*Chenopodium quinoa*) and lambsquarters (*Chenopodium album*). Plant Health Prog. doi: 10.1094/PHP-09-21-0121-RS

[B93] Urquizo F. E. L. Torres S. M. G. Tolonen T. Jaakkola M. Pena-Niebuhr M. G. von Wright A.. (2017). Development of a fermented quinoa-based beverage. Food Sci. Nutr. 5 (3), 602. doi: 10.1002/fsn3.436 28572947 PMC5448362

[B94] Vazquez P. Holguin G. Puente M. Lopez-Cortes A. Bashan Y. (2000). Phosphate-solubilizing microorganisms associated with the rhizosphere of mangroves in a semi-arid coastal lagoon. Biol. Fert. Soils. 30 (5), 460–468. doi: 10.1007/s003740050024

[B95] Vilcacundo R. Hernández-Ledesma B. (2017). Nutritional and biological value of quinoa (*Chenopodium quinoa* willd.). Curr. Opin. Food Sci. 14, 1–6. doi: 10.1016/j.cofs.2016.11.007

[B96] Vincent J. M. (1970). A manual for practical study of root nodule bacteria. IBP handbook no. 15 (Oxford: Black-well Scientific Publishers), 164.

[B97] Vishnupradeep R. Bruno L. B. Taj Z. Karthik C. Challabathula D. Kumar A.. (2022). Plant growth promoting bacteria improve growth and phytostabilization potential of *Zea mays* under chromium and drought stress by altering photosynthetic and antioxidant responses. Environ. Technol. Innov. 25, 102154. doi: 10.1016/j.eti.2021.102154

[B98] Walker N. Gupta R. Cheesbrough J. (2006). Blood pressure cuffs: friend or foe? J. Hosp. Inf. 63 (2), 167–169. doi: 10.1016/j.jhin.2005.10.019 16616799

[B99] Wang H. Inukai Y. Yamauchi A. (2006). Root development and nutrient uptake. Crit. Rev. Plant Sci. 25 (3), 279–301. doi: 10.1080/07352680600709917

[B100] Wang S. Walker R. Schicklberger M. Nico P. S. Fox P. M. Karaoz U.. (2021). Microbial phosphorus mobilization strategies across a natural nutrient limitation gradient and evidence for linkage with iron solubilization traits. Front. Microbiol. 12. doi: 10.3389/fmicb.2021.572212 PMC8261140 34248859

[B101] Wei Z. Hillier S. Gadd G. M. (2012). Biotransformation of manganese oxides by fungi: solubilization and production of manganese oxalate biominerals. Environ. Microbiol. 14 (7), 1744–1753. doi: 10.1111/j.1462-2920.2012.02776.x 22591055

[B102] Wei Y. Zhao Y. Shi M. Cao Z. Lu Q. Yang T.. (2018). Effect of organic acids production and bacterial community on the possible mechanism of phosphorus solubilization during composting with enriched phosphate-solubilizing bacteria inoculation. Bioresour. Technol. 247, 190–199. doi: 10.1016/j.biortech.2017.09.092 28950126

[B103] Wilson K. (2001). Preparation of genomic DNA from bacteria. Curr. Prot. Mol. Biol. 56 (1), 2–4. doi: 10.1002/0471142727.mb0204s56 18265184

[B104] Win K. T. Fukuyo T. Keiki O. Ohwaki Y. (2018). The ACC deaminase expressing endophyte pseudomonas spp. enhances NaCl stress tolerance by reducing stress-related ethylene production, resulting in improved growth, photosynthetic performance, and ionic balance in tomato plants. Plant Physiol. Biochem. 127, 599–607. doi: 10.1016/j.plaphy.2018.04.038 29730579

[B105] Xie H. Pasternak J. J. Glick B. R. (1996). Isolation and characterization of mutants of the plant growth-promoting rhizobacterium *Pseudomonas putida* GR12-2 that overproduce indoleacetic acid. Curr. Microbiol. 32, 67–71. doi: 10.1007/s002849900012

[B106] Yanez-Yazlle M. F. Romano-Armada N. Acreche M. M. Rajal V. B. Irazusta V. P. (2021). Halotolerant bacteria isolated from extreme environments induce seed germination and growth of chia (*Salvia hispanica* l.) and quinoa (*Chenopodium quinoa* willd.) under saline stress. Ecotoxicol. Environ. Saf. 218, 112273. doi: 10.1016/j.ecoenv.2021.112273 33940441

[B107] Yang A. Akhtar S. S. Fu Q. Naveed M. Iqbal S. Roitsch T.. (2020). *Burkholderia phytofirmans* PsJN stimulate growth and yield of quinoa under salinity stress. Plants 9 (6), 672. doi: 10.3390/plants9060672 PMC7355930 32466435

[B108] Yu P. Gutjahr C. Li C. Hochholdinger F. (2016). Genetic control of lateral root formation in cereals. Trends Plant Sci. 21, 951–961. doi: 10.1016/j.tplants.2016.07.011 27524642

[B109] Zaheer A. Malik A. Sher A. Mansoor Q. M. Mehmood A. Khan S. U.. (2019). Isolation, characterization, and effect of phosphate-zinc-solubilizing bacterial strains on chickpea (*Cicer arietinum* l.) growth. Saudi. J. Biol. Sci. 26, 1061–1067. doi: 10.1016/j.sjbs.2019.04.004 31303841 PMC6600776

[B110] Zahir Z. A. Shah M. K. Naveed M. Akhter M. J. (2010). Substrate-dependent auxin production by *Rhizobium phaseoli* improves the growth and yield of *Vigna radiata* l. under salt stress conditions. J. Microbiol. Biotechnol. 20, 1288–1294. doi: 10.4014/jmb.1002.02010 20890093

[B111] Zhang J. Wang P. Fang L. Zhang Q. A. Yan C. Chen J. (2017). Isolation and characterization of phosphate-solubilizing bacteria from mushroom residues and their effect on tomato plant growth promotion. Pol. J. Microbiol. 66, 57–65. doi: 10.5604/17331331.1234994 29359698

[B112] Zhan Y. Zhang Z. Ma T. Zhang X. Wang R. Liu Y.. (2021). Phosphorus excess changes rock phosphate solubilization level and bacterial community mediating phosphorus fractions mobilization during composting. Bioresour. Technol. 337, 125433. doi: 10.1016/j.biortech.2021.125433 34171708

