# Supplementary material for: Potential of mineral-solubilizing bacteria for physiology and growth promotion of Chenopodium quinoa Willd
Source: Front Plant Sci. 2022 Oct 10;13:1004833. doi: 10.3389/fpls.2022.1004833 (PMC9589155; doi:10.3389/fpls.2022.1004833)
Supplement: Supplementary file 1 [file Table_1.docx]

Supplementary Material

# Biochemical characterization

# Method: The Gram-straining of selected bacterial strains was performed by preparing the bacterial smears on glass slides, air-dried, and heat-fixed before staining (Walker, 2006). Safranine was used to cover the streak after decolonization with alcohol. Further, the smear was washed, and the slides were dried at room temperature. Smears were examined microscopically at 100X with an oil immersion objective to differentiate Gram-positive and Gram-negative bacteria. The bacterial strain's cell morphology, motility, and colony morphology were observed under a light microscope (Vincent, 1970).

**Supplementary Table S1.** Morphological characteristics of the bacterial isolates obtained from the rhizosphere of *Chenopodium quinoa*.

| **Isolate** | **Staining** | **Colony** | **Cell morphology** |
| --- | --- | --- | --- |
| *Bacillus altitudinis* Cq-3 | Gram-positive | Rod-shaped | Fuzzy white |
| *Pseudomonas flexibilis* Cq-32 | Gram-negative | Coccus | Reddish |
| *Bacillus pumilus* Cq-35 | Gram-positive | Rod-shaped | Yellow-orange |
| *Pseudomonas furukawaii* Cq-40 | Gram-negative | Rod-shaped | Orange |
| *Pontibacter lucknowensis* Cq-48 | Gram-positive | Rod-shaped | Yellow |
| *Ensifer* sp. Cq-51 | Gram-negative | Rod-shaped | Peach |

# References

Vincent, J. M. (1970). *A manual for practical study of root nodule bacteria. IBP handbook no. 15* (Oxford: Black-well Scientific Publishers), 164.

Walker, N., Gupta, R., and Cheesbrough, J. (2006). Blood pressure cuffs: friend or foe? *J. Hosp. Inf.* 63 (2), 167–169. doi: 10.1016/j.jhin.2005.10.019
